# Noncentrosymmetric Manganese Bismuth Thiocyanate Perovskites

**DOI:** 10.1021/acs.chemmater.6c02127

**Published:** 2026-10-08

**Authors:** Madeleine Geers, Dominik J. Kubicki, Stephen P. Argent, Jeremiah P. Tidey, Elodie Salager, Benjamin B. Duff, Oscar Fabelo, Laura Cañadillas-Delgado, Matthew J. Cliffe

**Affiliations:** † School of Chemistry, 6123 University of Nottingham, University Park, Nottingham NG7 2RD, U.K.; ‡ 56053 Institut Laue-Langevin, 71 avenue des Martyrs CS 20156, 38042 Grenoble Cedex 9, France; § Department of Chemistry—Ångström Laboratory, Uppsala University, Box 538, 751 21 Uppsala, Sweden; ∥ School of Chemistry, 1724 University of Birmingham, Birmingham B15 2TT, U.K.; ⊥ Department of Physics, 2707 University of Warwick, Gibbet Hill Road, Coventry CV4 7AL, U.K.; # CNRS, Université d’Orléans, CEMHTI UPR 3079, 1D avenue de la recherche scientifique, 45071 Orléans, France; ¶ RS2E, CNRS FR 3459, 15 rue Baudelocque, 80039 Amiens, France; ∇ Manchester Institute of Biotechnology, University of Manchester, Manchester M13 9PL, U.K.; ○ Department of Materials Science and Metallurgy, 2152 University of Cambridge, 27 Charles Babbage Road, Cambridge CB3 0FS, U.K.

## Abstract

Symmetry-lowering orderings in perovskites can produce a wide range of functions. Thiocyanate based perovskites, AM­(SCN)_3_, have orderings distinct from those of the atomic oxides, enabling new functional behavior. We report four new defect-ordered manganese thiocyanate perovskites K­{Mn­[Bi­(SCN)_6_]}, ${NH}_{4}\{Mn\left[Bi{\left(SCN\right)}_{6}]\right\}\cdot \frac{1}{8}H_{2}O$ , $Cs\{Mn\left[Bi{\left(SCN\right)}_{6}]\right\}\cdot \frac{1}{8}H_{2}O$ , and ${Gua}_{6}\{{Mn}_{9}{\left[Bi{\left(SCN\right)}_{6}\right]}_{8}\}\cdot 6H_{2}O$ , and solve their structures using a combination of powder X-ray, single crystal X-ray, single crystal electron and single-crystal neutron diffraction combined with solid-state ^1^H, ^13^C, and ^133^Cs MAS NMR. These frameworks each adopt a structure distinct from both previously reported thiocyanates, and other molecular and atomic perovskites. These noncentrosymmetric thiocyanate perovskites demonstrate how frustration between octahedral tilts and A-site ordering can produce complex function.

## Graphical abstract

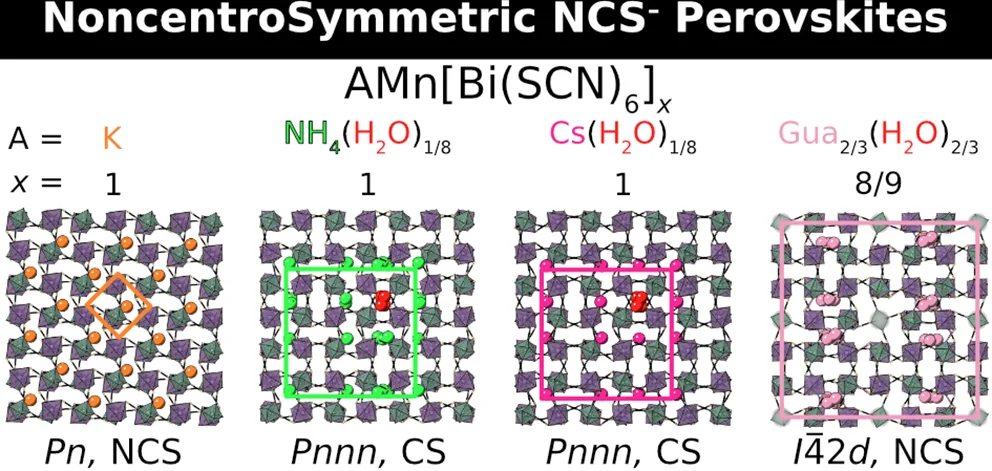

## Introduction

AMX_3_ perovskites are a playground for solid-state scientists. Their diversity of possible chemistries permits tailoring of compounds for specific physical properties.^1,2^ Engineering polar perovskites, to be used as ferroelectrics or for optical second harmonic generation, has been an enduring challenge, as the parent aristotype is nonpolar (Pm3̅m) , and most common symmetry-lowering distortions are not sufficient on their own to break the center of symmetry.^3^ Instead, the most technologically important polar perovskites, e.g. BaTiO_3_, rely on the off-centering of the M-site cation in its octahedral coordination environment due to the second-order Jahn–Teller distortion of the d^0^ transition metal.^4^ This mechanism is hard to generalize, and precludes other functional properties, particularly magnetism.^4,5^

Novel mechanisms for breaking the center of symmetry in perovskites are therefore of interest. Hybrid improper ferroelectricity, in which the combination of multiple symmetry-lowering distortions generates a new polarization, is a particularly exciting route, but is not common in oxides and mostly occurs in layered perovskite analogue oxides.^6^ Molecular perovskites, where molecules form one of the three components, are of particular interest as they offer novel ordering patterns which allow new mechanisms for polarity as well as outstanding structural diversity.^7,8^ Examples of center-of-symmetry breaking mechanisms include the ferroic ordering of polar A-site cations,^9^ unconventional tilts and shifts^8,10,11^ and the coupling of quadrupolar cations with octahedral tilting and ordered first-order Jahn–Teller distortions.^8,12^

Thiocyanate perovskites, A_*x*_[M­(SCN)_3_], are a growing family of hybrid materials (Figure 1).^13–17^ The distinct chemistry of the NCS^–^ termini means that all reported thiocyanate perovskites have rocksalt ordering of MS_6_ and M′N_6_ octahedra, with harder transition metals typically occupying the M′ site, and softer cations on the M site (M = Cd^2+^, Bi^3+^, Pt^4+^). The chemistry of the NCS^–^ ligand, in particular the preference of S for bent bond angles, means that all known thiocyanate perovskites have large octahedral tilts around all three axes.

**Figure 1:**
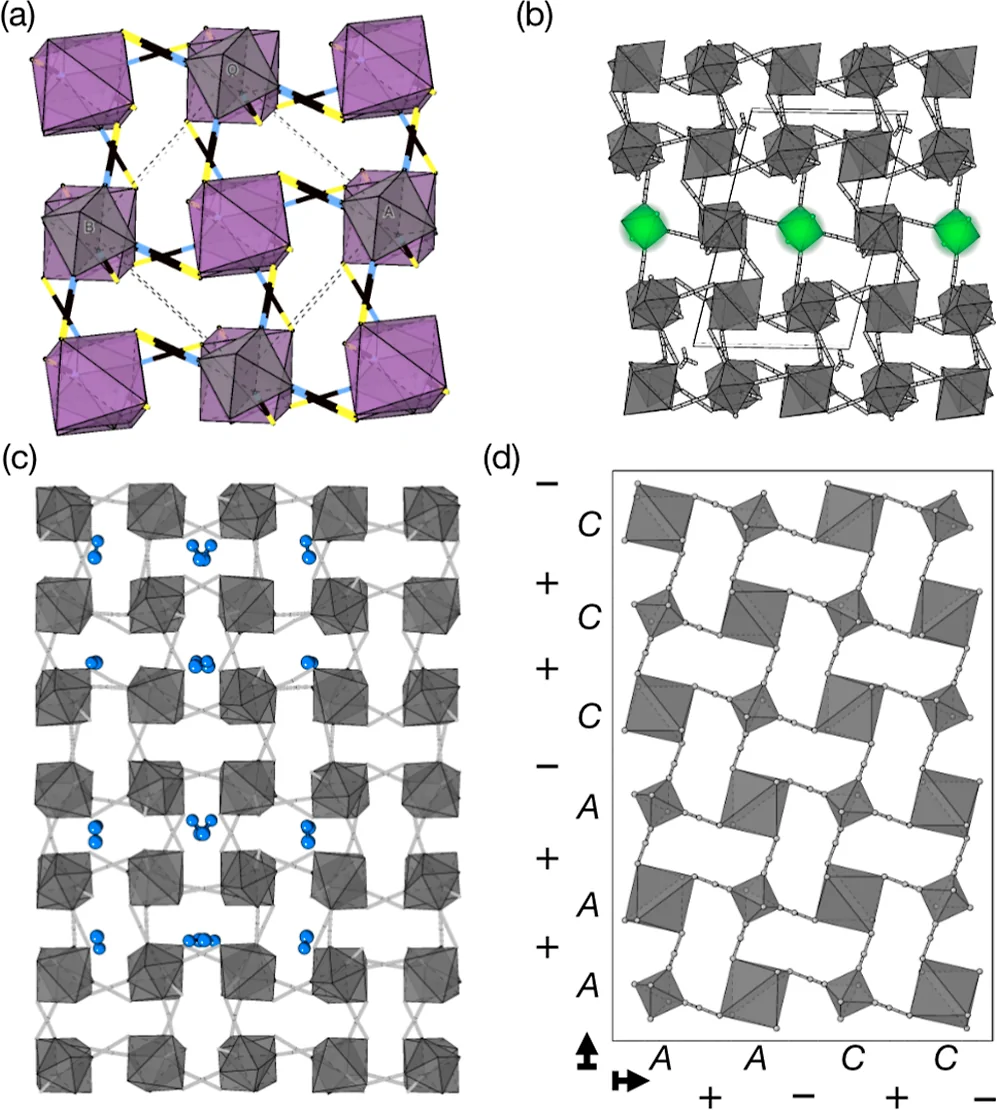
Thiocyanate perovskites with generic formula $A_{x}\{M{\left[M^{\prime }{\left(SCN\right)}_{6}\right]}_{1-y}\}\cdot z\left(guest\right)$ can have different orders. Each subpanel is illustrated by a representative compound given. (a) Rocksalt M-site order is always found in thiocyanate perovskites (Fe­[Bi­(SCN)_6_]).^18^ (b) Vacancy order is common in materials from aqueous synthesis or with large A sites $({\left(NH_{4}\right)}_{\frac{1}{2}}\left\{Ni{\left[Bi{\left(SCN\right)}_{6}\right]}_{\frac{5}{6}}\right\}$ ​ $\cdot 2.7H_{2}O)$ .^14^ (c) A-site cations are typically well-ordered and often in complex patterns ((CH_3_NH_3_)­{Ni­[Bi­(SCN)_6_]}).^13^ (d) Cooperative octahedral tilts are large and often complicated ((CH_3_NH_3_)­{Ni­[Bi­(SCN)_6_]}).^13^

In addition to this M-site order and octahedral tilting, some of us have previously reported that robust A-site ordering can be induced in vacancy-containing perovskites □A­{Ni­[Bi­(SCN)_6_]}, where □ is a vacancy site.^13^ The A-site order is coupled with the tilts, as tilting moves NCS^–^ ions from the edges of the pseudocubic cages into the center, and so the cations are positioned to avoid cages containing four NCS^–^ ions. In (CH_3_NH_3_)­{Ni­[Bi­(SCN)_6_]} the frustration inherent to the competition between A-site order and tilting leads to a complex pattern of octahedral tilts.^13^ This tilt pattern cannot be described by the conventional ‘Glazer’ notation, *a* ^*x*^ *b* ^*y*^ *c* ^*z*^, where *x*, *y* and *z* indicate whether the tilts in the plane normal to each axis are in phase (*x* = +), out of phase (*x* = −) or no tilt (*x* = 0), as consecutive planes along the *c* axis alternate between being in and out of phase (*z* = +−), and along the *b* axis are in phase, in phase then out of phase (*y* = ++−), giving a tilt pattern of *a* ^+^ *b* ^++–^ *c* ^+–^ in an extended notation. This complex tilt pattern thus results in an A-site ordering where the cations form a block of 3 × 2 × 1 pseudocubic cages. These new orderings in thiocyanate perovskites offer new mechanisms for polarity.^13,14^ Nevertheless, there are no reported polar thiocyanate perovskites.

In this paper we report four new thiocyanate perovskites of target composition □A­{Mn­[Bi­(SCN)_6_]}, A = K^+^, Cs^+^, ${NH}_{4}^{+}$ , and $C{{\left({NH}_{2}\right)}_{3}}^{+}$ (guanidinium, Gua^+^). We use a combination of X-ray, neutron and electron diffraction with solid-state magic angle spinning NMR (MAS NMR) to determine their structures, and show that these materials also possess the combination of M-site rocksalt order, octahedral tilts and A-site vacancy order, with none of these materials being isostructural with a known perovskite. We find that two materials, A = K^+^ and Gua^+^, are noncentrosymmetric, with this symmetry breaking arising from the hybrid coupling of octahedral tilts and cation order.

## Results

### [□K]­Mn­{Bi­(SCN)_6_}

We began by synthesizing the example with the smallest cation, **KMnBi**. From a butanone solution we obtained an orange microcrystalline powder, which powder X-ray diffraction measurements demonstrated to be a crystalline material with a perovskite-like structure. We attempted to fit the diffraction data using the known thiocyanate perovskite structures, [□A]­Ni­{Bi­(SCN)_6_}, A = K, CH_3_NH_3_, Gua, and □_2_FeBi​(SCN)_6_,^13,18^ but these were not able to account for all the observed peaks, suggesting a new combination of orderings was likely.

We therefore attempted to grow a single crystal through slow evaporation recrystallizations from distilled H_2_O or butanone, but did not produce crystals large enough for single crystal X-ray diffraction measurements. We then turned to an alternative method of diffraction to determine the structure of **KMnBi**, single crystal electron diffraction. From this powder sample we collected data sets on a range of nanocrystals at room temperature using a Rigaku XtaLAB Synergy-ED electron diffractometer.

The data were indexed and integrated using CrysAlisPro version 1.171.44.144a,^19^ and four appropriate data sets were scaled and merged to produce a single composite data set. The structure was solved using SHELXT and refined using olex2.refine as implemented in Olex2 in the kinematic approximation. **KMnBi** crystallizes in monoclinic polar *Pn*, *a* = 8.715(3) Å, *b* = 8.551(3) Å, *c* = 12.005(4) Å, β = 90.25(3)° (Figure 2c) (Table 1, Section S4).^20–22^

**Figure 2:**
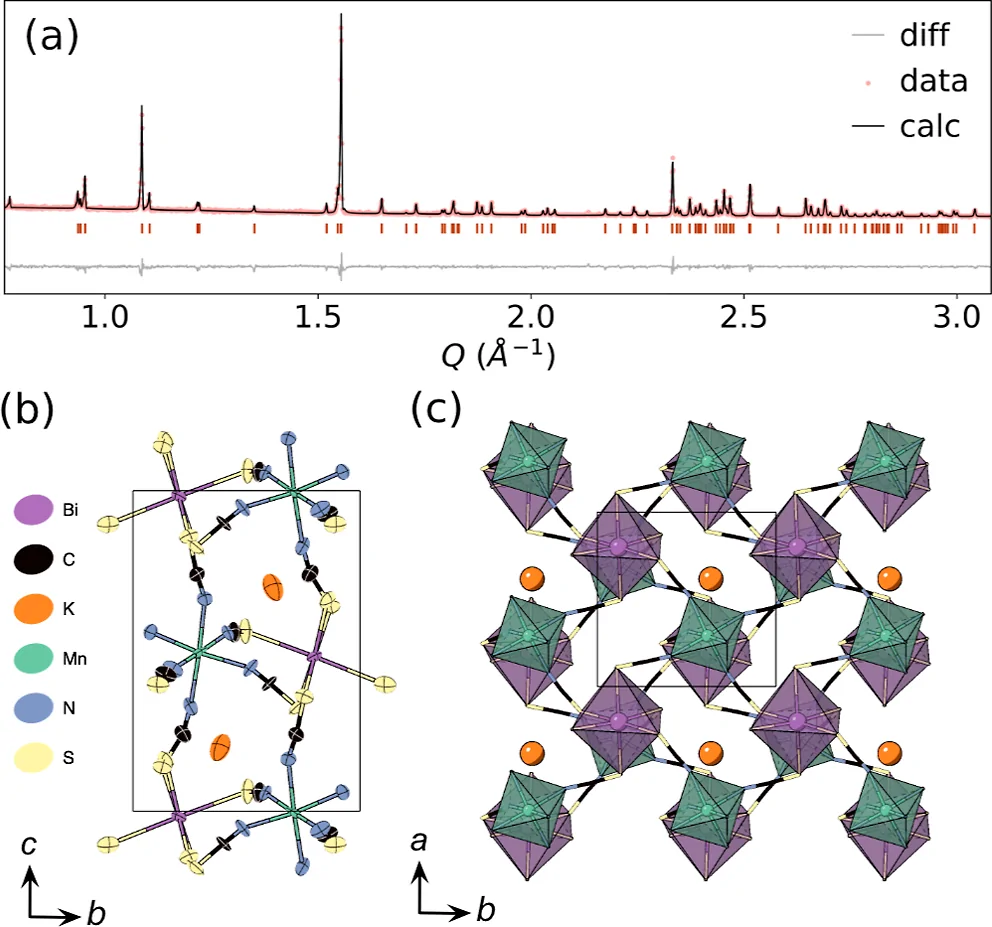
**KMnBi** structure (crystallizes in *Pn*). (a) Le Bail refinement of the powder X-ray diffraction data. (b) ORTEP of the asymmetric unit from electron diffraction. (c) The structure viewed along the *c* axis.

**Table 1:** Selected Crystallographic and Refinement Parameters for Each Single Crystal Diffraction Measurement^a^

compound	**KMnBi**	**CsMnBi**	**CsMnBi**	**CsMnBi**-postheat
chemical formula	KMnBiN_6_C_6_S_6_	Cs_8_Mn_8_Bi_8_N_48_C_48_S_48_O	Cs_8_Mn_8_Bi_8_N_48_C_48_S_48_O	Cs_8_Mn_8_Bi_8_N_48_C_48_S_48_
radiation type	electron	neutron	Ag Kα X-rays	Ag Kα X-rays
λ (Å)	0.0251	1.45947	0.56088	0.56089
temperature (K)	293(5)	80(2)	293(2)	293(2)
crystal system	monoclinic	orthorhombic	orthorhombic	orthorhombic
space group	*Pn*	*Pnnn*	*Pnnn*	*Pnnn*
*a* (Å)	8.715(3)	12.3953(4)	12.4386(4)	12.4386(3)
*b* (Å)	8.551(3)	24.6320(7)	24.7803(7)	24.7803(8)
*c* (Å)	12.005(4)	24.6136(7)	24.8038(7)	24.8038(8)
β (°)	90.25(3)	90	90	90
*V* (Å^3^)	894.6(6)	7515.0(4)	7645.3(4)	7645.3(4)
*M*_r_ (g mol^–1^)	651.52	5978.6	5978.6	5962.3
crystal size (mm)	ca. 0.001 × 0.001 × 0.001	4 × 3 × 2	0.1 × 0.08 × 0.05	0.1 × 0.08 × 0.05
*R*[*F* ^2^ > 2σ (*F* ^2^)]	0.131	0.251	0.0610	0.067
w*R*(*F* ^2^)	0.316	0.267	0.081	0.223
*S*	1.04	8.86	2.13	1.03

compound	**CsMnBi**	**NH**_**4**_ **MnBi**	**NH**_**4**_ **MnBi**	**GuaMnBi**
chemical formula	C_48_Bi_8_Cs_8_Mn_8_N_48_S_48_	Mn_8_Bi_8_N_56_C_48_S_48_O	Mn_8_Bi_8_N_56_C_48_S_48_OH_28_	Mn_9_Bi_8_N_72_C_56_S_48_O_6_H_48_
radiation type	Ag Kα X-rays	Mo Kα X-rays	neutron	Mo Kα X-rays
λ (Å)	0.56089	0.7107	1.45818	0.7107
temperature (K)	400(2)	120(2)	20(2)	120(2)
crystal system	orthorhombic	orthorhombic	orthorhombic	tetragonal
space group	*Pnnn*	*Pnnn*	*Pnnn*	*I*4̅2*d*
*a* (Å)	12.452(4)	12.4172(3)	12.442(3)	37.0950(4)
*b* (Å)	24.936(9)	24.4213(6)	24.422(3)	37.0950(4)
*c* (Å)	24.859(9)	24.5217(7)	24.3705(17)	24.4182(4)
β (°)	90	90	90	90
*V* (Å^3^)	7719(5)	7436.1(3)	7405(2)	33600.4(9)
*M*_r_ (g mol^–1^)	5962.3	5027.28	5055.4	5530.84
crystal size (mm)	0.1 × 0.08 × 0.05	0.14 × 0.07 × 0.03	3 × 3 × 1	0.13 × 0.11 × 0.09
*R*[*F* ^2^ > 2σ (*F* ^2^)]	0.091	0.084	0.160	0.055
w*R*(*F* ^2^)	0.169	0.102	0.161	0.135
*S*	3.10	2.37	3.12	1.10

a *S* = goodness of fit. For complete refinement statistics see Tables S2–S5 in the Supporting Information.

Having obtained a structural model from single crystal electron diffraction, we were able to fit our powder X-ray diffraction data to verify the structure of the bulk microcrystalline sample and improve the precision of the unit cell parameters. Le Bail refinements were performed, refining freely the unit cell parameters (*R*_wp_ = 26.0%, χ^2^ = 2.31). The refined unit cell parameters, *a* = 8.8098(2) Å, *b* = 8.6138(1) Å, *c* = 12.1219(2) Å, β = 90.365(1)°, have the expected level of agreement with the electron diffraction data (Figure 2a).

In **KMnBi** the M-site cations have rocksalt ordering, with Mn octahedrally coordinated to the nitrogen termini and Bi octahedrally coordinated to the sulfurs (Figure 2b). The edges of the pseudocubic cage are the [11̅0], [110], and [001] directions. The K^+^ A-site cations occupy half the cages, with each filled cage surrounded by empty cages, that is, with rocksalt ordering (Figure 2c). Along the [11̅0] and [110] directions, the octahedra alternate their sense of rotation, and along [001] all the octahedra rotate in the same direction, i.e. *a* ^–^ *a* ^–^ *c* ^+^ Glazer tilt sequence.^3^

Polar perovskites are comparatively rare, and hence to confirm that **KMnBi** adopts a polar structure, we carried out symmetry analysis using the ISOTROPY Software Suite.^23^ We first used FINDSYM to search for any potential higher-symmetry space groups that could describe the structure, which confirmed that no additional symmetry operators were present. We also carried out symmetry analysis of a double-double perovskite with atomic ligands, AA′MM′X_6_ and the same tilt sequence and cation orderings, i.e. possessing *a* ^–^ *a* ^–^ *c* ^+^ tilts, rocksalt A-site and rocksalt M site orderings. This analysis, as previously reported,^24^ gives the same *Pn* space group as we find for **KMnBi**, showing the polarity emerges from the tilts and cation order.

### $\left[{{□}_{\frac{7}{8}}Cs\left(H_{2}O\right)}_{\frac{1}{8}}\right]Mn\left\{Bi{\left(SCN\right)}_{6}\right\}$

Having discovered a new structure type for K^+^, we therefore explored a larger cation, Cs^+^, as previous reports suggest that this is a fruitful method to induce more complex ordering patterns.^13,16^

We grew large orange cuboid single crystals of **CsMnBi** through slow evaporation in acetone (up to 4 mm linear dimension). Single crystal X-ray diffraction (SCXRD, 293 K) was carried out to determine the structure. The Bragg reflections were indexed in the orthorhombic space group *Pnnn* with the unit cell *a* = 12.4386(4) Å, *b* = 24.7803(7) Å, *c* = 24.8038(7) Å (Figure 3, Table 1). As is typical for thiocyanate perovskites, there is strict rocksalt M-site order with [MnN_6_] and [BiS_6_] octahedra. Two of the 12 crystallographically independent sulfur atoms are split over two sites, with refined occupancies of 0.66(10):0.34(10) (S11a/S11b) and 0.65(11):0.35(11) (S12a/S12b), a feature commonly found in these materials.^13^ The octahedral tilt sequence is *a* ^+–^ *b* ^+–^ *c* ^+^ in the extended Glazer notation ([CCAA]­[CCAA]­[C] in Peel notation, where the tilts in each layer are described as clockwise, C, or anticlockwise, A).^13^ Examination of the symmetry of the structure using ISODISTORT confirmed that the tilt system in combination with the M-site order determines the structural symmetry: the A-site order does not further break the structural symmetry.^23^

**Figure 3:**
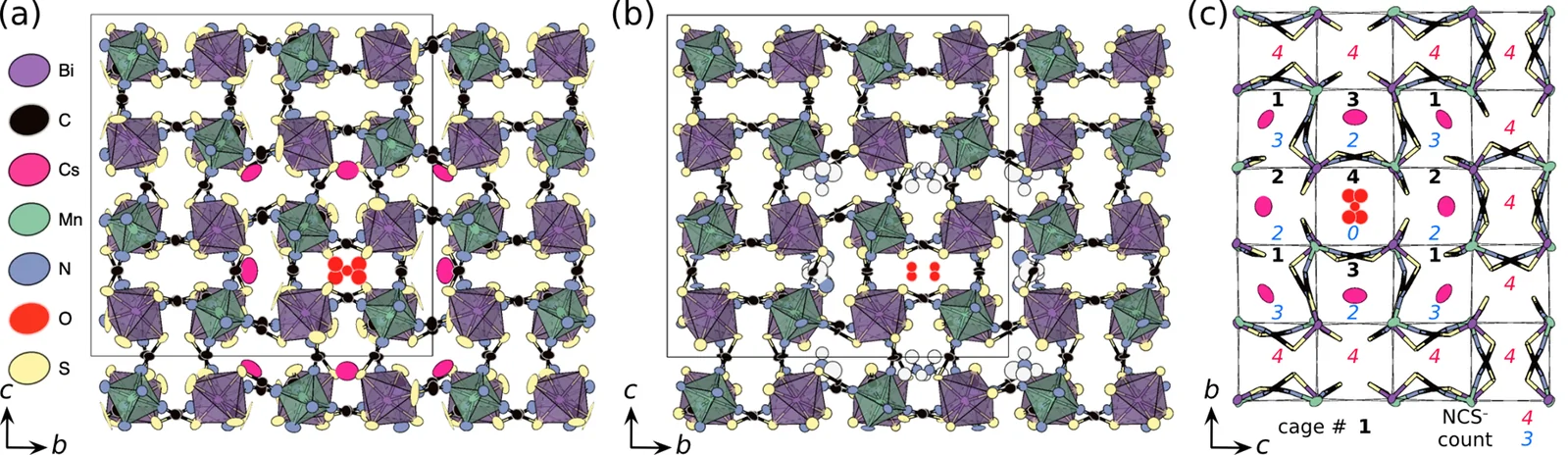
Neutron-derived ORTEP figures of (a) **CsMnBi** and (b) **NH**_**4**_ **MnBi** viewed along the *a* axis. (c) Structure of a single layer of **CsMnBi** showing the cage numbering (bold) and NCS count within each cage (italics).

The A-sites order in 3 × 3 × 1 blocks, surrounded by empty cages. These guest-free cages have four thiocyanates pointing into the cavity. There are four distinct occupied A-site cages which we number in ascending complexity and disorder (Figures 3 and 4). Cage 1 is occupied by a single, ordered Cs^+^ cation (Cs1). Cage 2 is occupied by two Cs^+^ cations with an occupancy of 0.5 separated from each other by 0.705(5) Å. Cage 3 is occupied by single Cs^+^ cations with a large oblate atomic displacement parameters (ADPs) suggesting significant positional disorder. Cages 1, 2, and 3 together give the crystal a Cs/Mn ratio of 1:1, the expected stoichiometry. Nevertheless, some weak residual features could be identified in Cage 4.

**Figure 4:**
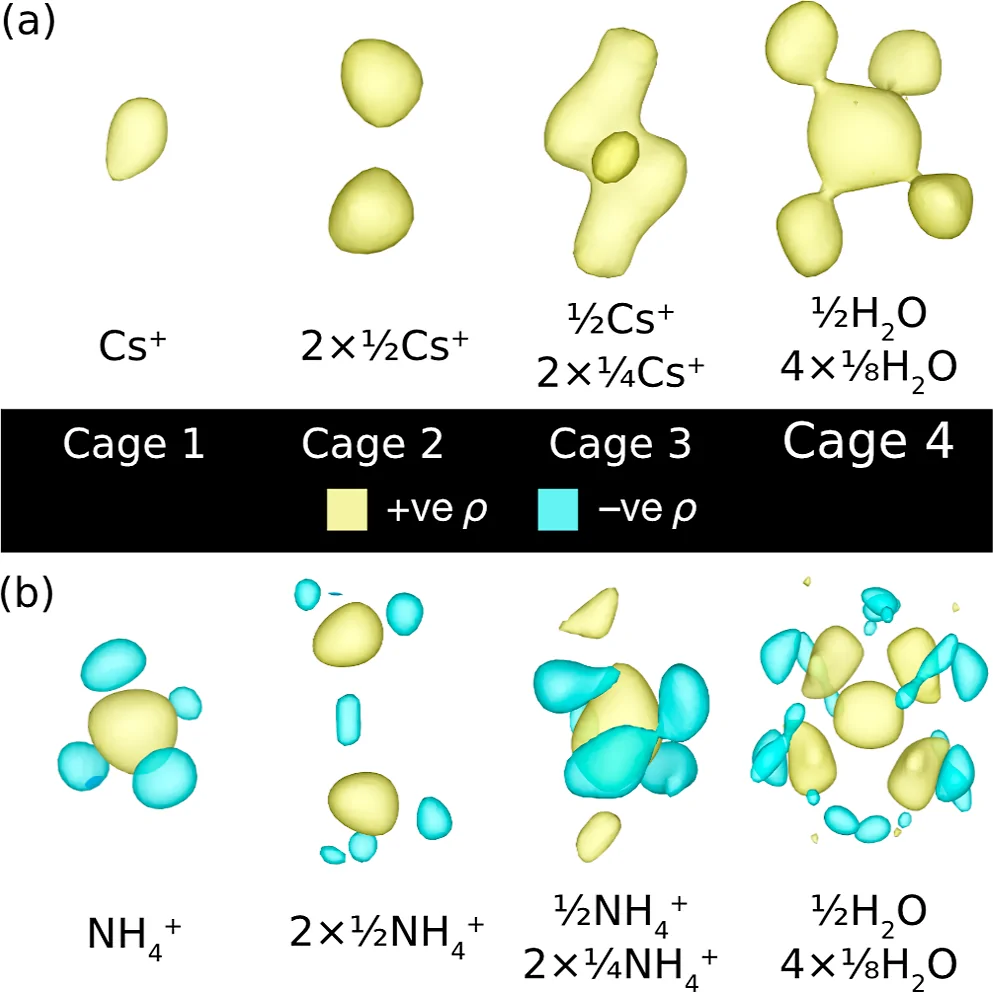
Fourier difference scattering density maps before guest atoms were added for each of the four occupied cages from neutron diffraction for (a) **CsMnBi** and (b) **NH**_**4**_ **MnBi**.

These peaks were too weak to be Cs^+^ cations, and as water and other neutral guest species have previously been found in thiocyanate perovskites, we proceeded with this hypothesis.^14,18^ We refined a central oxygen atom with varying occupancies: either refining freely (χ^2^ = 3.00), fixed with an occupancy of 0.5 (χ^2^ = 2.90), or a fixed occupancy of 1 (χ^2^ = 2.86), our preferred model.

To assess the stability of this hydrate, we determined the structure of a single crystal of **CsMnBi** at 400 K using SCXRD, and then repeated the data collection after cooling to 293 K. We found that, although the framework and Cs^+^ cations are essentially unchanged, the electron density relating to the water in Cage 4 is no longer observed at 400 K, and is also not present at 293 K postheat treatment, indicating that heating to 400 K dehydrates the compound.

To provide more information on the light atoms in the structure we therefore carried out single crystal neutron diffraction measurements using the D19 diffractometer at the ILL on a large single crystal (4 × 3 × 2 mm^3^).^25^ The structure was measured at 80 K to minimize the effect of dynamic disorder. This structure was largely unchanged from the higher temperature X-ray structures, though the reduction in motion at low temperature combined with the additional contrast provided by the negative scattering length of the ^1^H nuclei did allow identification of residual scattering density from the water hydrogen atoms supporting our assignment of water in Cage 4.

Significant cation and guest disorder suggested that a closer examination of the local structure would be valuable. We therefore carried out solid-state MAS NMR measurements, measuring ^133^Cs, ^13^C, and ^1^H NMR spectra (Figure 5). The room-temperature ^133^Cs MAS NMR spectrum of **CsMnBi** shows five distinct cesium local environments at approximately 450, 150, 50, 5, and −295 ppm with total integrated intensity in the ratio 0.3:1:1:2:3. Considering that the diamagnetic ^133^Cs shift range is typically approximately 0–400 ppm, this result suggests that the peaks at 450 and −295 ppm experience hyperfine coupling to the Mn sites, likely through a pseudocontact (dipole–dipole, through-space) interaction. Given that the crystallographic structure contains only three Cs sites, the spectrum indicates that the local structure is more complex than suggested by the average structure. This complexity can also be seen in the Fourier density maps which show multiple peaks (Figure 4), as well as the large ADPs even at low temperatures. To determine which Cs sites are affected by the paramagnetism of the unpaired Mn 3d electrons, we carried out variable-temperature ^133^Cs MAS NMR measurements at 4.7 T and 40 kHz, which resulted in sideband-free spectra spanning the range from approximately −500 to +600 ppm. While the positions of the peaks in the diamagnetic range are not appreciably affected by temperature, the positions of the hyperfine-shifted peaks are strongly temperature dependent. This is most clearly seen for the peak at −295 ppm at 300 K, which shifts to −350 ppm at 253 K, corresponding to an average apparent temperature coefficient of +1.3 ppm/K. Fitting the temperature-dependent shifts to the Curie law gives Δ­(*T*) = 40.7 – (9.98 × 10^4^/*T*), with an excellent fit (*R* ^2^ = 0.995), indicating that this temperature dependence in shift is likely due to isolated paramagnetism. The sizes of the paramagnetic shifts are considerably smaller than that of CsMn­[Co­(CN)_6_] (+566 ppm), which is ascribed to a more direct contact shift.^26^

**Figure 5:**
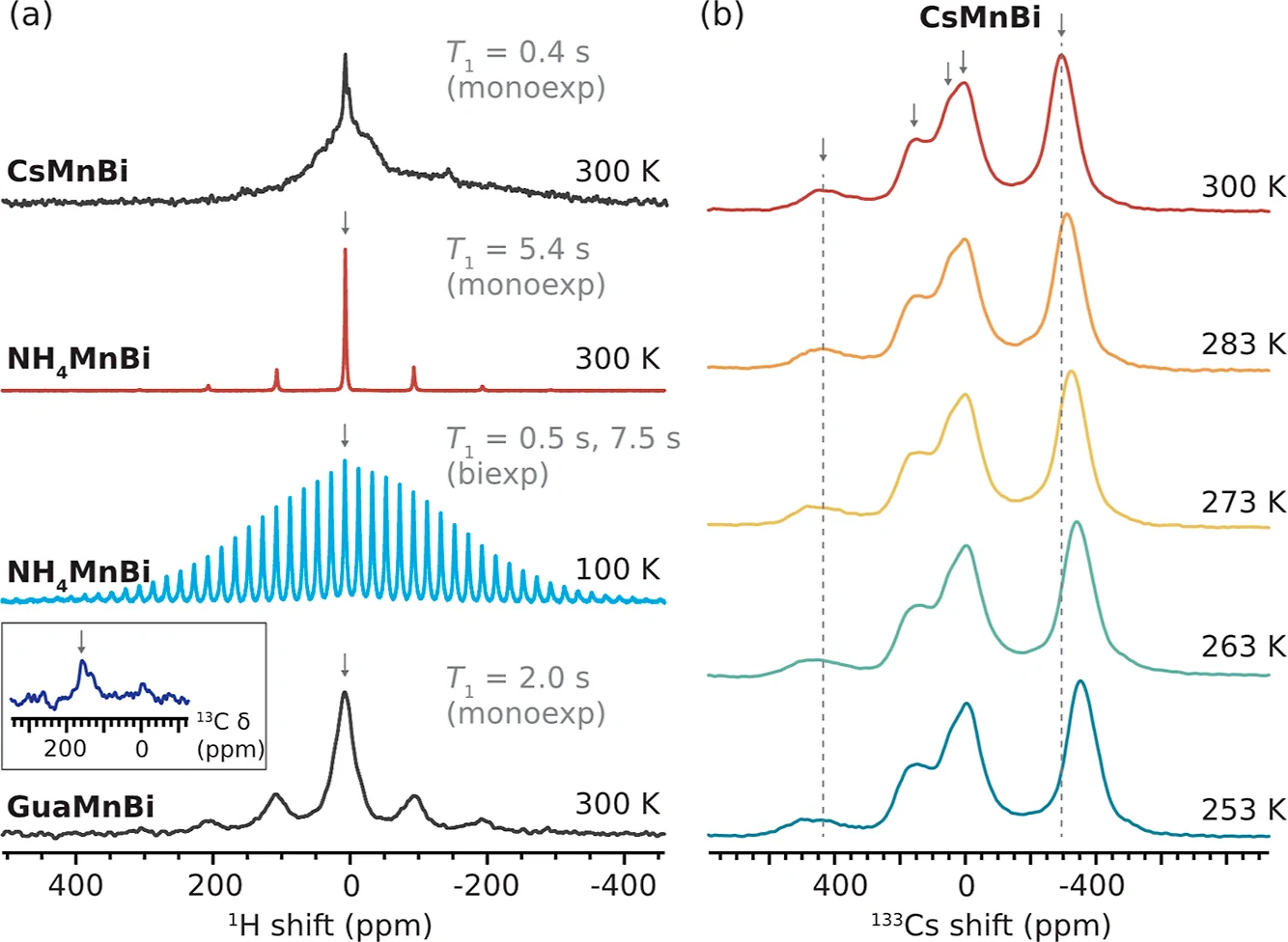
Solid-state MAS NMR characterization. (a) Echo-detected ^1^H of **CsMnBi** (4.7 T, 30 kHz MAS, 300 K); **NH**_**4**_ **MnBi** (4.7 T, 20 kHz MAS, 300 K); **NH**_**4**_ **MnBi** (9.4 T, 8 kHz MAS, 100 K), and **GuaMnBi** (4.7 T, 20 kHz MAS, 300 K); (b) echo-detected variable-temperature ^133^Cs of **CsMnBi** in the 253–300 K range (4.7 T, 40 kHz MAS). The fitted ratio of the five Cs sites is 0.3:1:1:2:3. Where multiple peaks are visible, isotropic peaks are indicated by arrows. The inset in panel (a) shows the echo-detected ^13^C MAS NMR spectrum of **GuaMnBi**. For further details and acquisition parameters see Table S1.

The ^1^H MAS NMR spectrum of **CsMnBi** shows a broad component spanning approximately −200 to +100 ppm, together with two sharp components at 1 and 6 ppm. We attribute these signals to hydration water at different distances from the paramagnetic center, with water molecules closest to Mn exhibiting the largest paramagnetic broadening. This result is consistent with the neutron diffraction measurements, where small amounts of water were also detected.

### $\left[{□}_{\frac{7}{8}}{\left(NH_{4}\right)\left(H_{2}O\right)}_{\frac{1}{8}}\right]Mn\left\{Bi{\left(SCN\right)}_{6}\right\}$

Having investigated the largest stable monovalent atomic cation, we focused on the smallest molecular cation, NH_4_^+^. We obtained from slow evaporation in butanone or acetone dark red-orange cuboid single crystals (100–200 μm length) and carried out single crystal X-ray diffraction (120 K) to determine the structure.

The integrated reflections suggested a structure with tetragonal *P*4_2_/*mnm* symmetry and a unit cell of *a* = *b* = 24.4649(5) Å, *c* = 12.4173(3) Å (standard tetragonal setting), or state that the orthorhombic axes are kept. However, this structure could not be stably refined, so we solved and refined the framework structure in *P*1 to identify the framework symmetry, which revealed that **NH**_**4**_ **MnBi** also crystallized in the orthorhombic *Pnnn* space group (Table 1, *a* = 12.4172(3) Å, *b* = 24.4213(6) Å, *c* = 24.5217(7) Å). We therefore integrated, solved and refined the structure in *Pnnn*. Examination of the framework structure revealed it to be analogous to that of **CsMnBi**, with rocksalt M-site order, and an *a* ^+–^ *b* ^+–^ *c* ^+^ tilt sequence (Figure 3b). To find the guest NH_4_^+^ and any H_2_O molecules we carried out single crystal neutron diffraction at 20 K (D19, Institut Laue-Langevin, 3 × 3 × 1 mm^3^).^27^

The 20 K neutron diffraction data showed the same symmetry as the X-ray structure (*Pnnn*). We were able to solve the structure and refine the framework and clearly locate the guests. Having found these guests in the neutron scattering map, we were then able to locate the guests in the residual electron density map determined from refinement of the framework model against X-ray data (Figure 4). As in **CsMnBi** there are four symmetry independent occupied cages in a 3 × 3 × 1 block, with the surrounding cages vacant (Figure 3). As found in **CsMnBi**, the larger the number of thiocyanates in a cage, the more ordered the guests (Figure 4). We have used the same cage numbering for both structures (increasing number indicates increasing disorder).

From the low temperature neutron diffraction data, it was evident that Cage 1 contains an ordered, fully occupied ammonium cation (N13, H1–4), with positive scattering density in the central position and four discrete positions of negative scattering density corresponding to the negative scattering length of ^1^H. Cage 2 also contains ammonium, with a central atom surrounded by negative scattering density peaks (N14, H5–8). The scattering density is spread over a larger area, both for nitrogen and hydrogen positions, suggesting positional and/or orientational disorder of the ammonium ions. There are two symmetry-equivalent NH_4_^+^ positions within Cage 2, with a N···N separation of 2.51(4) Å, less than the sum of van der Waals radii (3.10 Å), so both positions could not be simultaneously occupied.^28^ As freely refined occupancy was half within error, 0.58(7), we fixed the cation occupancy to 0.5. In addition, the H–N–H bond angles and N–H distances were restrained.

Cage 3 has three positive peaks (Figure 4). The central peak is surrounded by four broad negative scattering peaks and hence is assigned to be NH_4_^+^ (N15, H9–12). The two smaller positive peaks are symmetry-related and very close to the central atom, *d*(N15···N16) = 1.41(8) Å, and to each other, *d*(N16···N16) = 2.51(15) Å. Both smaller peaks are surrounded by broad negative scattering density from ^1^H. The central atom, N15, has an occupancy of 0.5, and the two symmetry-related outer atoms, N16, have an occupancy of 0.25, giving a total cage occupancy of one NH_4_^+^. We located and refined the H atoms surrounding N15, but despite the presence of negative scattering density, we could not locate and refine all seven expected hydrogen atoms. We were able to locate and refine one H atom (H10) with a N–H distance restraint and the subsequent three H atoms could be refined using a riding model with tetrahedral geometry.

Cages 1, 2, and 3 together charge balance the framework completely. However, there is still significant residual neutron scattering density within Cage 4. This density is broad, suggesting positional and orientational disorder. The four largest positive peaks are symmetry-related and have distances of 0.61(9), 0.79(6), and 0.96(7) Å between them and therefore cannot be simultaneously occupied. The sites could be refined stably with a total occupancy of 1 with either oxygen (*R*_obs_ = 14.76%) or nitrogen (*R*_obs_ = 14.88%), indicating this is likely water. There is also significant negative scattering density surrounding these sites, indicative of hydrogen, but the breadth of the density precluded the stable location of any H atoms. The site is accordingly modeled as a single disordered water molecule.

Our neutron and X-ray diffraction analysis strongly suggests the framework contains no larger molecule than ammonium or water; however, incorporation of solvent or other guests is not unprecedented in molecular perovskites. The residual scattering density for both X-ray and neutron diffraction did not show any evidence of the crystallization solvent (for neutron-sized crystals, butanone; for X-ray-sized crystals, acetone) even as a disordered species.

The ^1^H MAS NMR spectrum of **NH**_**4**_ **MnBi** shows a single narrow peak centered at 6.8 ppm (full width at half-maximum, fwhm, of 2.6 ppm), as expected for NH_4_^+^ (Figure 5). Surprisingly, this peak is not appreciably affected by either hyperfine interactions, as indicated by the absence of a pseudocontact shift, or paramagnetic relaxation enhancement (PRE), as evidenced by a relatively long ^1^H *T*_1_ of 5.4 s. We attribute this result to the relatively large distance between the NH_4_^+^ ions and the Mn centers, which vary from 3.77 to 4.59 Å, based on the neutron diffraction structure.

Our diffraction experiments showed clear evidence of hydration water, refined as $\frac{1}{8}H_{2}O$ per NH_4_^+^. There is no evidence of any additional signals in the room-temperature spectrum, which is most likely related to rapid proton exchange between NH_4_^+^ and H_2_O, leading to an averaged chemical shift. We attempted to resolve the two species using cryogenic MAS at 100 K and found that the NH_4_^+^ peak shifts slightly to 7.4 ppm and broadens to a fwhm of 4.1 ppm relative to room temperature. However, there is still no evidence for a separate H_2_O species. We attribute this discrepancy to two possible explanations: (a) H_2_O experiences strong PRE and/or a large hyperfine shift because it is closer to, or possibly bound to, the Mn center, and is therefore not preserved in the 100 K echo at 8 kHz owing to its short *T*_2_; or (b) the H_2_O peak overlaps with the broadened NH_4_^+^ peak. We note that at 100 K the ^1^H *T*_1_ becomes biexponential, with two components, weighted approximately 1:1, of 0.5 and 7.5 s. This indicates that NH_4_^+^ is not strongly affected by the PRE even at 100 K, with a slightly more compact cell (coefficient of volume thermal expansion between 120 and 20 K is +42 MK^–1^). We also note the extensive spinning sideband manifold, which we attribute to bulk magnetic susceptibility effects rather than pseudocontact shift anisotropy, since there is no appreciable pseudocontact shift contribution to the experimentally observed isotropic ^1^H shift.

For both **CsMnBi** and **NH**_**4**_ **MnBi** we have thus determined that Cages 1, 2, and 3 contain a single cation, increasing in disorder from Cage 1 through 3, and Cage 4 contains a highly disordered water molecule.

### ${\left[{□}_{3}{Gua}_{6}\right]{Mn}_{9}\left\{Bi{\left(SCN\right)}_{6}\right\}}_{8}\cdot 6H_{2}O$

We finally explored the effect of using a larger cation, guanidinium, which we have previously found to form complex, vacancy-containing thiocyanate perovskites.^13^ Slow evaporation from butanone yielded dark red-orange, cuboid single crystals (100–200 μm length). We carried out single crystal X-ray diffraction measurements at 120 K to determine the structure of ${Gua}_{6}{Mn}_{9}{\left[Bi{\left(SCN\right)}_{6}\right]}_{8}\cdot 6H_{2}O$ , **GuaMnBi**.

The phase was found to exhibit weak superstructure reflections at $h=\frac{1}{2}$ , $k=\frac{1}{2}$ , $l=\frac{1}{2}$ , and was solved in the tetragonal space group *I*4̅2*d* (*a* = *b* = 37.0950(4) Å, *c* = 24.4182(4) Å). We note this is a noncentrosymmetric space group, both piezoelectric and optically active, for which the corresponding centrosymmetric minimal supergroups (either *I*4_1_/*amd* or *I*4_1_/*acd*) have additional systematic reflection conditions (e.g., for *hk*0 reflections *h*, *k* = 2*n* must hold) and so can be ruled out without requiring refinement of the structure.

The framework, like other thiocyanate perovskites, shows rocksalt ordering of the M-sites (Table 1, Figure 6). There are additionally ${\left[Bi{\left(SCN\right)}_{6}\right]}^{3-}$ vacancies, common in more complex thiocyanate perovskites containing bismuth.^13,14^ One-ninth of all ${\left[Bi{\left(SCN\right)}_{6}\right]}^{3-}$ are missing and these *V*_Bi_ form a 3 × 3 order in the *ab* plane. The coordination spheres of the Mn ions adjacent to the vacancies are completed by H_2_O molecules. The voids created by the vacancies are large enough that they would have significant solvent-accessible volumes of 140 and 103 Å^3^. The electron density difference map showed a number of diffuse residual electron density features at the center of each void which could not be refined as atoms. We thus used a solvent mask as implemented in Olex2 ^21^ to account for this difficult to model density. The total electron count was consistent with one Gua^+^ cation per void containing 32 and 29 e^–^ each, compared to 32 e^–^ for Gua^+^.

**Figure 6:**
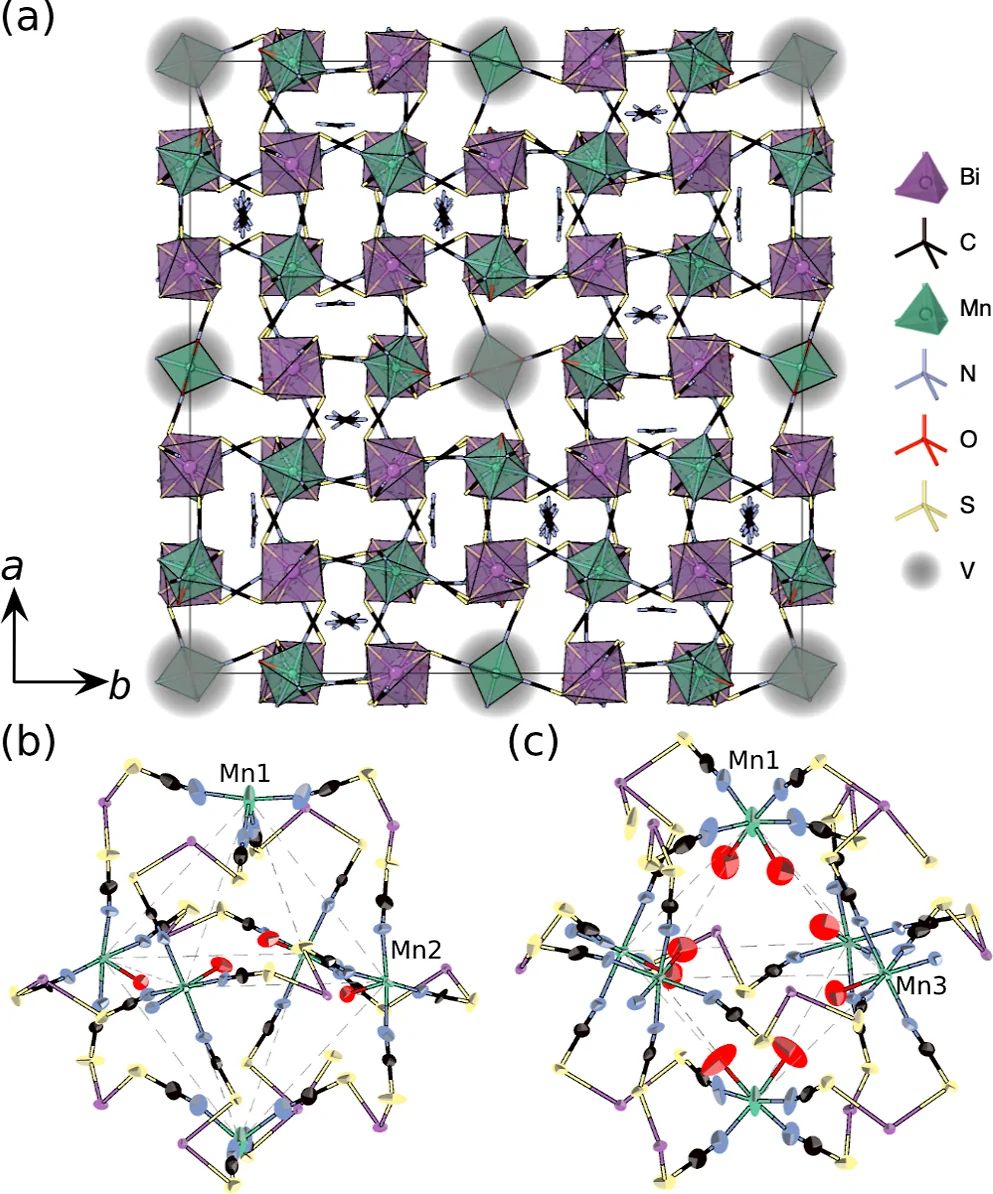
(a) The structure of **GuaMnBi** viewed along the *c* axis with only a single plane of cages shown. Voids are marked with diffuse gray spheres. The structures of the framework surrounding the voids are shown as ORTEP plots for the voids at (b) (0, 0, 0) and (c) (0.5, 0.5, 0). The *cis* coordination of the MnN_4_O_2_ coordination for Mn1, Mn2, and Mn3 can be seen in both cages.

In the complete pseudocubic cages, we were able to identify two crystallographically distinct Gua cations, one ordered and one disordered over two distinct general positions, which lie approximately within {101} type planes. The total formula is thus $\left[{□}_{3}{Gua}_{6}\right]{Mn}_{9}{\left\{Bi{\left(SCN\right)}_{6}\right\}}_{8}\cdot 6H_{2}O$ , consistent with the expected Mn­(II) oxidation state.

The complexity of the structure is also reflected in its octahedral tilts. Two-thirds of the *bc* and *ac* planes (which are related by symmetry) have continuous thiocyanate connectivity (without any vacancies), and within those planes there are standard cooperative tilts. In the third plane, there are Mn atoms with vacancies on both sides (Mn1) and have a ${\left[Mn{\left(NCS\right)}_{4}{\left({OH}_{2}\right)}_{2}\right]}^{2-}$ coordination environment (Figure 6b,c). Surprisingly, this octahedrally coordinated Mn ion is not *trans*, as would be expected from the perovskite connectivity, but *cis*, and so is not accurately described as a tilt. The tilts along the *c* axis, neglecting octahedra disconnected by vacancies, are in phase (i.e., are *c* ^+^ or C), as in other examples. However, the tilts along the *a* and *b* axes are unusual, and adopt the *a* ^+–+–++^ *b* ^+–+–++^ *c* ^+^ tilt sequence (CCCCAA) for the two-thirds of metals that have continuous connectivity. The one-third that are disrupted have the Peel tilt sequence (.CC.AA) (Figure 6). The A-site cation order consists of 2 × 1 × 1 blocks that connect at the corners with other blocks. As in other thiocyanate perovskites, we find that the A-site cations strictly avoid cages containing four thiocyanate ligands.

In the ^1^H MAS NMR spectrum of **GuaMnBi**, we observe a broad peak centered at 7.8 ppm, with a fwhm of 35.9 ppm, consistent with the presence of Gua^+^ (Figure 5). The relatively long ^1^H *T*_1_ of 2 s suggests that Gua does not experience appreciable PRE and its chemical shift suggests no significant influence of pseudocontact shifts. The inset in Figure 5 shows the corresponding ^13^C MAS NMR spectrum with two relatively broad (fwhm approximately 15 ppm) peaks at 157 ppm, corresponding to guanidinium ions not affected by hyperfine effects, and 137 ppm, which we attribute to guanidinium ions experiencing a slight pseudocontact shift of approximately 20 ppm. These may correspond to guanidinium in cages and in defect sites, though definitive assignment is impossible. The NMR data, together with the X-ray diffraction data, paint a picture of a complex structure in which the guests pattern the tilt sequence.

### Magnetic Properties

Because **KMnBi** and **GuaMnBi** are noncentrosymmetric, we investigated whether they order magnetically and are hence potentially multiferroic, particularly as Cr­[Bi­(SCN)_6_] magnetically orders.^29^ We measured the bulk magnetic susceptibility of **KMnBi**, **NH**_**4**_ **MnBi**, and **GuaMnBi** as representatives of the three structure types and the isothermal magnetization at 2 K for the noncentrosymmetric **KMnBi** and **GuaMnBi** (Figures 7, S4 and S5).

**Figure 7:**
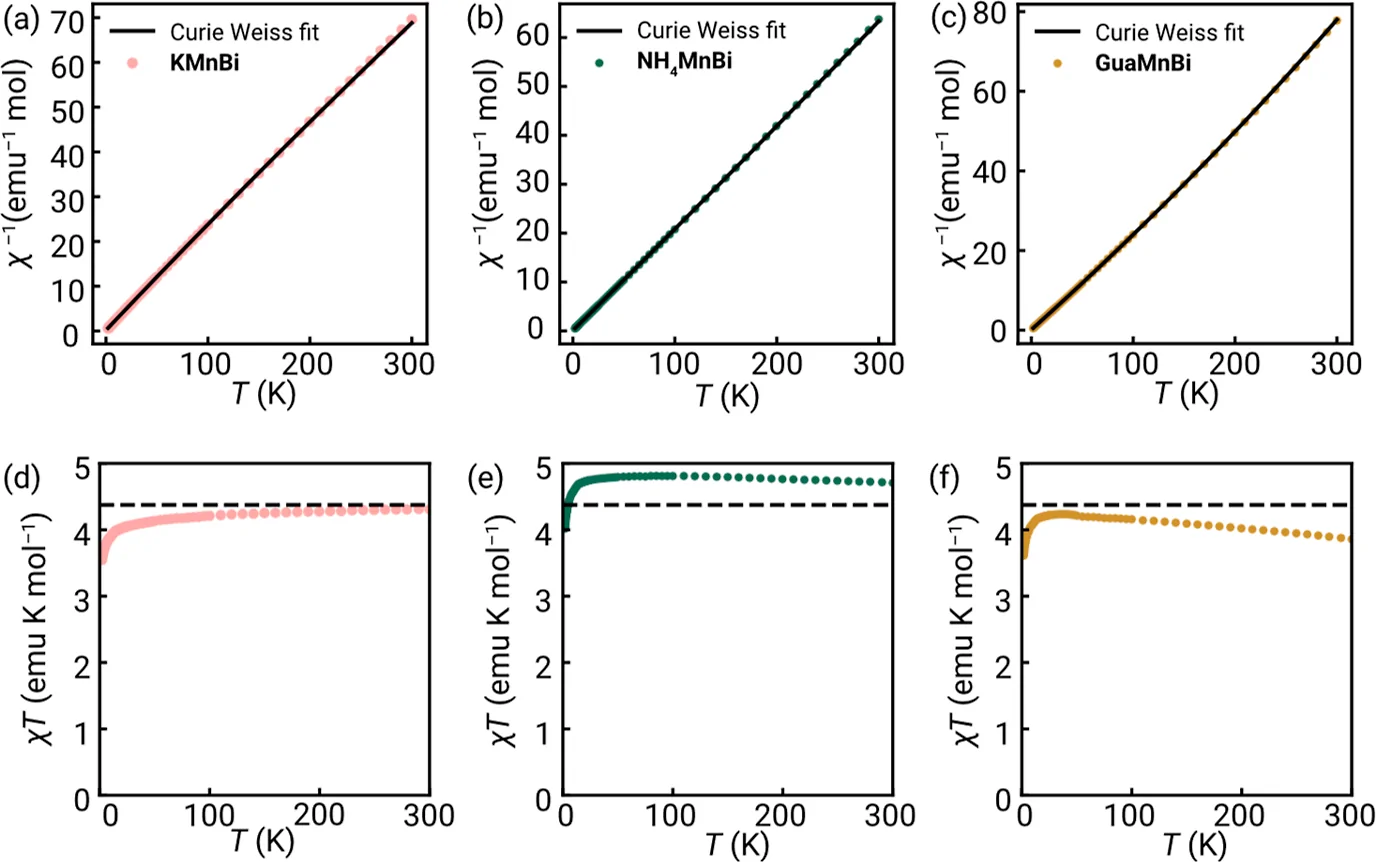
Inverse magnetic susceptibility measurements as a function of temperature, χ^–1^(*T*), with an applied field of 0.01 T for (a) **KMnBi**, (b) **NH**_**4**_ **MnBi** and (c) **GuaMnBi**. Curie–Weiss fit with additional temperature-independent susceptibility term shown as black line. Magnetic susceptibility temperature product as a function of temperature χ*T*(*T*), (d) **KMnBi**, (e) **NH**_**4**_ **MnBi** and (f) **GuaMnBi**.

None of the three measured compounds showed evidence of magnetic order above 2 K. Curie–Weiss fitting, including a temperature-independent susceptibility term, showed that the magnetic interactions were very weak with Curie–Weiss temperatures, |*T*_CW_| < 1 K (Table 2). The Curie constant was consistent with high spin $S=\frac{5}{2}$ Mn^2+^ ions (Table 2), with deviations likely due to small mass errors and diamagnetic corrections.

**Table 2:** Curie–Weiss Fit Parameters for **KMnBi**, **NH**_**4**_ **MnBi**, and **GuaMnBi**

compound	*T*_CW_ (K)	*g*	μ_eff_ (μ_B_)
**KMnBi**	–0.8(2)	1.9(1)	5.8(2)
**NH**_**4**_ **MnBi**	–0.6(2)	2.1(1)	6.2(3)
**GuaMnBi**	–0.6(2)	2.0(1)	5.9(3)

There is no observed hysteresis in the isothermal magnetization data measured at 2 K for either **KMnBi** or **GuaMnBi**, and the moments are saturated by μ_0_ *H* = 5 T (Figure S4). The data obey the Brillouin function for isolated $S=\frac{5}{2}$ using the *g* values determined from Curie–Weiss fitting, further confirming that any interactions are very weak.

## Discussion

Using a combination of single crystal X-ray, neutron and electron diffraction, we have solved the structures of four vacancy-ordered thiocyanate perovskites, $A_{x}{\{Mn\left[Bi{\left(SCN\right)}_{6}\right]}_{y}\}$ ​ $\cdot zH_{2}O$ , with A = K^+^, NH_4_^+^, Cs^+^, Gua^+^, and *x* ≈ 1, *y* ≈ 1, and $0\leq z\leq \frac{2}{3}$ . The M-sites are rocksalt ordered, as is uniformly found in thiocyanate perovskites.^13–18,30^ This ordering is driven by the segregation between the soft S and hard N termini, and means thiocyanates are well-ordered even in monometallic frameworks.^31–33^ This hard–soft acid base mechanism is distinct from the difference in cation charge which underlies charge ordering in most atomic perovskites.^24^

Although these new structures are closely related to both each other and previous perovskites, all four possess new structure types with a novel combination of A-site order and octahedral tilts (Figure 8). A-site ordering is unusual in itself in perovskites and where it does occur, layering is most common, as this minimizes strain on the M-site octahedra,^24^ as in ${□}_{\frac{2}{3}}{\mathrm{Ln}}_{\frac{1}{3}}{TaO}_{3}$ (Ln = lanthanides, La to Yb),^34^ and ${□}_{\frac{2}{3}}{La}_{\frac{1}{3}}{NbO}_{3}$ .^35^ Layered A-site order has not yet been found in any thiocyanate perovskite.

**Figure 8:**
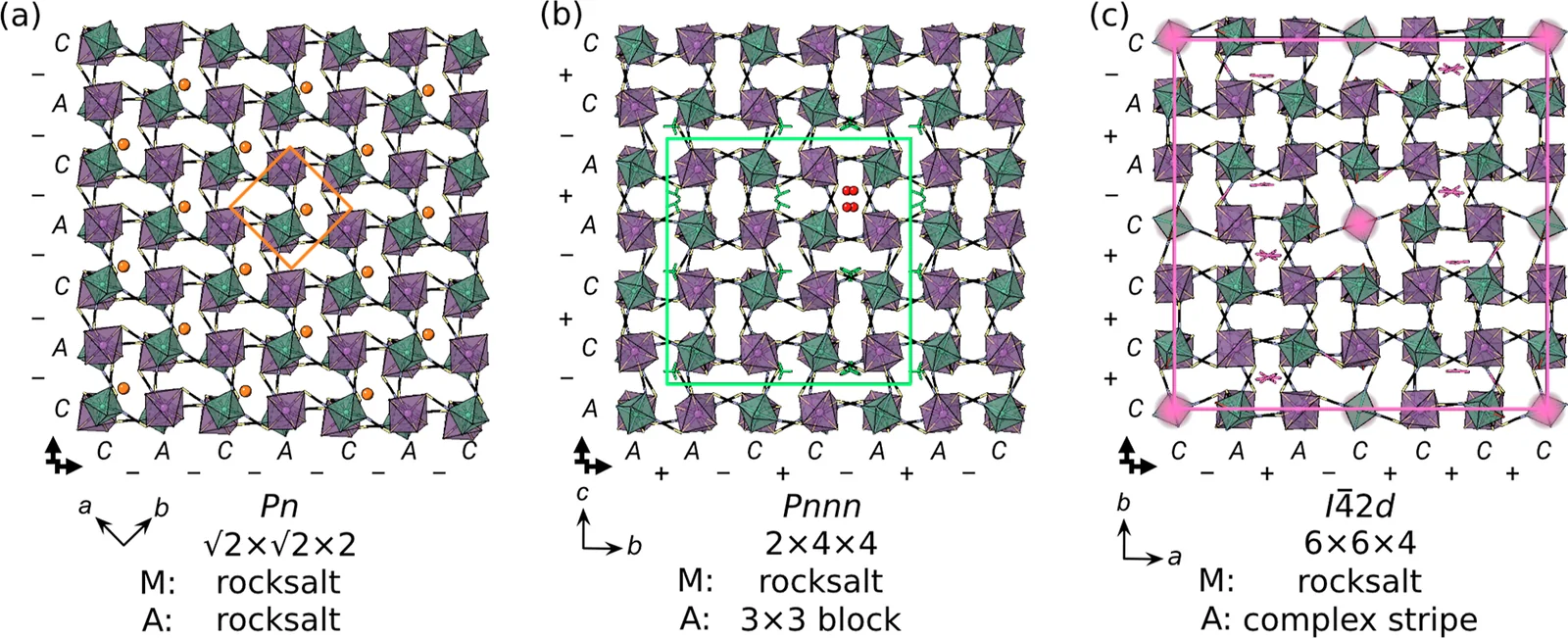
Summary of the structures and their orderings for (a) **KMnBi** along *c*, (b) **NH**_**4**_ **MnBi** along *a*, which is the same as **CsMnBi** and (c) **GuaMnBi** along *c*. Supercells are given relative to the hypothetical *Pm*3̅*m* aristotype, and all structures have in-phase tilts along the viewing axis.

**KMnBi** has rocksalt A-site order. This is known only for two fluoride perovskites, which both have rocksalt M-site order and no octahedral tilts, leading to a cubic *F*4̅3*m* symmetry.^36,37^ When combined with the common *a* ^–^ *a* ^–^ *c* ^+^ tilt sequence, this A- and M-site rocksalt ordered structure is predicted to be lowered to the polar *Pn* space group.^38^ **KMnBi** has exactly this tilt sequence and cation ordering pattern and does crystallize in *Pn* as predicted by the irreducible representation analysis. The nickel analogue, K­{Ni­[Bi­(SCN)_6_]}, has the same M-site order and tilt sequence, but instead has columnar A-site order leading to a nonpolar structure.^13^ These different A-site orderings are predicted to be similar in energy for K­{Ni­[Bi­(SCN)_6_]}. The phase selection for **KMnBi** is likely to be equally finely balanced, so it is very plausible that the difference between Mn­(II) and Ni­(II) could produce different A-site orderings.

The three larger cations produce A-site orderings which have not previously been reported. **NH**_**4**_ **MnBi** and **CsMnBi** both have 3 × 3 × 1 blocks, containing eight cations and neutral guests. Block ordering is unusual, having only been reported for [□CH_3_NH_3_]­Ni­{Bi­(SCN)_6_}, which contains fully occupied 3 × 2 × 1 blocks.^13^ We have previously introduced an empirical rule for predicting the A-site ordering from the tilt pattern: if there are cages with four thiocyanates, there will be no A-site guests and the fewer thiocyanates in a cage, the more disordered the A-site species is likely to be. These rules of thumb also hold true for the Mn thiocyanate perovskites we report here. The 3 × 3 × 1 block structure found for **NH**_**4**_ **MnBi** and **CsMnBi** is entirely consistent with the *a* ^+–^ *b* ^+–^ *c* ^+^ tilt sequence: $\frac{9}{16}$ of all cages have fewer than four thiocyanates, with $\frac{4}{16}$ cages containing three, $\frac{4}{16}$ containing two and $\frac{1}{16}$ containing no thiocyanates. This complex ordering is necessary as all classical zone-corner Glazer tilt-systems have every cage containing three thiocyanates, other than *a* ^+^ *a* ^+^ *a* ^+^, which has $\frac{3}{4}$ cages containing four thiocyanates and $\frac{1}{4}$ containing no thiocyanates, incompatible with 1:1 A-site/vacancy ratios.

The $\frac{9}{16}$ ratio of cages containing fewer than four thiocyanates, with an extra, more spacious, cage, provides an explanation for why these hydrate phases crystallize in this complex structure. It is less clear, however, why these compounds crystallize as hydrates. Water uptake has been reported in A-site vacant □_2_Cr­{Bi­(SCN)_6_}, which can hydrate until half the A-sites are occupied by water, [□(H_2_O)]­Cr­{Bi­(SCN)_6_}.^18^ K^+^- and NH_4_^+^-containing materials are typically isostructural due to their similar sizes,^39,40^ as found in hybrid frameworks including metal formates^41^ and even other thiocyanate perovskites.^13^ NH_4_^+^ is a strong H-bond donor and so will likely interact more strongly with water than K^+^, perhaps suggesting why a hydrate phase is preferred for **NH**_**4**_ **MnBi**, and hence why the 3 × 3 × 1 block structure forms. However, Cs^+^ is typically more weakly interacting with water than K^+^, and the cations and water molecules do not directly interact in **NH**_**4**_ **MnBi** or **CsMnBi**, cutting against this line of reasoning. Further, the 3 × 2 × 1 block structure has previously been found to accommodate the even larger CH_3_NH_3_^+^ cation, with strict A­[MBi­(SCN)_6_] stoichiometry and no additional guests: showing that size alone cannot be the driving force for this complex structure. It perhaps suggests that the greater flexibility of the MnN_6_ octahedra due to the absence of crystal field stabilization favors the 3 × 3 × 1 block structure.

Frameworks containing significant metal or ligand vacancies typically have high guest solvent content, notably the Prussian Blue analogues,^42,43^ though these voids have also been observed in highly defective thiocyanate perovskites^14^ and ${Gua}_{3}\{{Ni}_{6}{\left[Bi{\left(SCN\right)}_{6}\right]}_{5}\}$ .^13^

**GuaMnBi** has the most complex structure of any thiocyanate perovskite. It can be compared to the equivalent Ni phase, which has the comparatively simple *a* ^+^ *a* ^+^ *a* ^+^ tilt sequence with conventional quadruple-perovskite 3:1 A-site order. However, this Ni­(II) phase has a deceptively complex structure, as it is both defective, containing ${\left[Bi{\left(SCN\right)}_{6}\right]}^{3-}$ vacancies, and there are weak peaks indicating incommensurate order that could not be readily modeled.^13^ This combination of ${\left[Bi{\left(SCN\right)}_{6}\right]}^{3-}$ vacancies and complex framework order is also observed in **GuaMnBi**. **GuaMnBi** can also be fruitfully compared to the defective thiocyanate perovskites synthesized in aqueous H_3_[Bi­(SCN)_6_], which contain extensive hydrogen-bonded water and metal site vacancies. Although the unit cell of **GuaMnBi** is very large (*V* = 33600.4 Å^3^), we expect that even more complex thiocyanate perovskites could exist. This complexity means that structure solution of new thiocyanate perovskites from powder diffraction data, which is typical for oxides, might be very challenging, so single crystal data, whether neutron, X-ray or electron will be essential.

Although these Gua^+^ containing thiocyanate perovskites are both defective, other Gua^+^ hybrid perovskites are not ordinarily defective, e.g. GuaM­(HCOO)_3_ (M = Mn, Fe, Co, Ni, Cu, Zn)^12^ and $GuaMn{\left(H_{2}POO\right)}_{3}$ ,^44^ though it is possible to use aliovalent doping to introduce M-site vacancies.^45,46^

Hybrid perovskites containing large, doubly charged molecular cations have A-site orderings that approach the complexity of thiocyanate perovskites, for example $\left[DAB\right]{Mn}_{2}{\left(H_{2}POO\right)}_{6}$ , DAB = 1,4-diazabicyclo[2.2.2]­octane-1,4-diium,^44^ and the family [TPC*n*TP]­Mn_2_(NCNCN)_6_, TPC*n*TP = ${\left[{\left(C_{3}H_{7}\right)}_{3}N\left({CH}_{2}\right)nN{\left(C_{3}H_{7}\right)}_{3}\right]}^{2+}$ , *n* = 4, 5.^47^ The TPC*n*TP dications in [TPC*n*TP]­Mn_2_(NCNCN)_6_ are large enough to span two different cages, ordering with a head-to-tail or herringbone pattern depending on cation size.^47^ The DAB cations in $\left[DAB\right]{Mn}_{2}{\left(H_{2}POO\right)}_{6}$ order instead into (111) type layers separated by empty cages. Hydrogen bonding between DAB and hypophosphite is likely the driving force in this structure, but interestingly, all vacant pseudocubic cages contain the hypophosphite anion, in a similar fashion to thiocyanate perovskites.

**KMnBi** and **GuaMnBi** are noncentrosymmetric thiocyanate perovskites. The mechanism for their noncentrosymmetry relies on the combination of A-site order, M-site order and octahedral tilting. This is in contrast to other polar hybrid perovskites, in which noncentrosymmetry is produced by the ordering of (quadru)­polar molecular A-site cations, e.g. $\left[{\left({CH}_{3}\right)}_{2}{NH}_{2}\right]Zn{\left(HCOO\right)}_{3}$ ,^48^ or unconventional (noncooperative) tilts, e.g. ${NH}_{4}Cd{\left({HCO}_{2}\right)}_{3}$ ^8,10^ or 6-azaspiro[5.5]­undecane­[Cd­(NCNCN)_3_].^11^ **KMnBi** crystallizes in the monoclinic space group *Pn*. The *a* ^–^ *a* ^–^ *c* ^+^ tilt sequence is the most commonly reported pattern in atomic perovskites,^24^ and is also observed in perovskites with molecular ligands including AMn­(HCOO)_3_ (A = CH_3_NH_3_^+^ and (CH_2_)_3_NH_2_^+^).^49–51^ Double perovskites, AA′MM′X_6_, with the *a* ^–^ *a* ^–^ *c* ^+^ tilt sequence, M-site rocksalt ordering and the absence of A-site order, adopt the centrosymmetric *P*2_1_/*n* symmetry^52^ (e.g. La_2_LiSbO_6_ ^53^ and Sr_2_NdBiO_6_ ^54^). In **KMnBi** the addition of A-site rocksalt ordering breaks the center of symmetry, lowering the symmetry to *Pn*. Ferroelectricity is possible by symmetry; however, the most obvious mechanisms for switching are unwinding very large tilts or changes in A-site cation order, which are likely to have large activation barriers.

Although **GuaMnBi** has a very complex ordering of A-site cations and guests, it is the framework itself which breaks the centrosymmetry, as it possesses the same symmetry as the total structure. If displacements of the M-site cations are neglected, the M-site cation substructure can be described by a $\frac{1}{\sqrt{2}}\times \frac{1}{\sqrt{2}}\times \frac{1}{2}$ cell with *I*4/*mmm* symmetry, which is the Laue class of the most intense reflections in the diffraction pattern. The complex octahedral tilt pattern, together with the metal displacements, is therefore responsible for the symmetry-breaking distortion from *I*4/*mmm* to *I*4̅2*d*.

These MnBi perovskites, despite the lack of centrosymmetry and paramagnetic ions, are not likely to be platforms for multiferroic behavior as the superexchange is very weak, as has been seen in nonperovskite MnBi­(SCN)_5_.^55^ This does not rule out interesting very low temperature behavior, or indeed higher temperature behavior in other metal thiocyanates, which can have liquid nitrogen temperature magnetic order.^33^

## Conclusion

We have reported the synthesis and crystal structures for four manganese­(II) bismuth­(III) thiocyanate frameworks. The complexity of these structures meant that in addition to single crystal X-ray diffraction it was necessary to use neutron diffraction for **NH**_**4**_ **MnBi** and **CsMnBi** where we obtained mm^3^-sized single crystals; electron diffraction for **KMnBi**, where we did not obtain large enough crystals for SCXRD analysis; and NMR measurements to resolve guest behavior in **CsMnBi**, **NH**_**4**_ **MnBi**, and **GuaMnBi**.

Despite the chemical similarities between Mn and Ni, these compounds are not isostructural to their Ni analogues. In particular the combination of the A-site cation incorporated and the octahedral tilt sequence produces in each case, to the best of our knowledge, an unreported perovskite structure type. **NH**_**4**_ **MnBi**, **CsMnBi**, and **GuaMnBi** exhibit new octahedral tilt sequences and new A-site ordering patterns and **KMnBi** is the first known double-double rocksalt perovskite with octahedral tilting. Although these structures are complex, it is possible using thiocyanate cage occupancy to predict the A-site ordering pattern for the nondefective perovskites.

Perhaps most excitingly, these complex orders produce the first thiocyanate perovskites with polarity, demonstrating the utility of generating frustration through including large A-site cations in vacancy ordered thiocyanate perovskites. The robust coupling between A-site order and octahedral tilts is a powerful tool; however, the ability to rationally predict and hence design the tilt and A-site order pairing remains an open challenge as the balance of energetics between ordering patterns is subtle. Further, vacancy patterns cannot yet be easily rationalized. We anticipate that synthetic exploration of thiocyanate perovskites, even those with changes anticipated to generate near isostructural analogues e.g. Mn^2+^ for Ni^2+^ or NH_4_^+^ for K^+^, is likely to find many new orderings, and hence new dielectric function.

## Methods

### Synthesis

$Bi{\left({NO}_{3}\right)}_{3}\cdot 5H_{2}O$ (485 mg, 1 mmol), KNCS (583 mg, 6 mmol) and $Mn{\left({NO}_{3}\right)}_{2}\cdot 4H_{2}O$ (251 mg, 1 mmol) were stirred overnight in butanone (5 mL), filtered, and the solvent removed *in vacuo* to give **KMnBi**. **CsMnBi**, **NH**_**4**_ **MnBi**, and **GuaMnBi** were prepared analogously from CsNCS, NH_4_NCS, and GuaNCS. Single crystals grew by slow evaporation over 2–7 months (Section S1).

### Diffraction

Powder X-ray data for **KMnBi** (Cu Kα) were fitted in FullProf.^56^ Electron diffraction data for **KMnBi** were collected at room temperature on a Rigaku XtaLAB Synergy-ED (200 kV) and processed in CrysAlisPro.^19^ The structure was solved with SHELXT^20^ and refined kinematically in olex2.refine.^21,22^ Single-crystal X-ray data were collected for **NH**_**4**_ **MnBi** and **GuaMnBi** at 120 K (Mo Kα, Rigaku SuperNova) and for **CsMnBi** at 293 K, at 400 K and at 293 K after heating (Ag Kα, Bruker D8 Venture). Neutron data were collected on D19 (ILL, λ = 1.458 Å) for **NH**_**4**_ **MnBi** at 20 K^27^ and **CsMnBi** at 80 K.^25^ The X-ray and neutron structures were solved with SHELXT or SUPERFLIP.^57^ **GuaMnBi** (an inversion twin, refined with a solvent mask) and postheating **CsMnBi** were refined in SHELXL,^58^ and the rest in Jana2006.^59^ Full details are given in Sections S2–S5 and Tables S2–S5.

### Solid-State NMR

Echo-detected ^1^H, ^13^C, and ^133^Cs MAS NMR spectra were recorded at 4.7 T (Bruker Avance III, 1.3 or 4 mm rotors, 8–40 kHz MAS). ^1^H spectra of **NH**_**4**_ **MnBi** were recorded at 100 K and 9.4 T (3.2 mm LTMAS probe, 8 kHz). Shifts were referenced to adamantane; acquisition parameters are in Section S6 and Table S1.

### Magnetometry

Susceptibility (2–300 K, 0.01 T) and isothermal magnetization (2 K, ±5 T) were measured on a Quantum Design MPMS3 and corrected for diamagnetism (Section S7).^60^

## Supplementary Material



## Data Availability

Additional research data for this article may be accessed at no charge and under a CC-BY license at the University of Cambridge Repository, DOI: 10.17863/CAM.134724 and Zenodo, DOI: 10.5281/zenodo.23197280

## Notes

Additional experimental details for synthesis, powder X-ray, single-crystal X-ray, 3D electron and neutron diffraction, solid-state NMR and magnetometry; crystallographic tables (PDF)
