# Supplementary material for: Noncentrosymmetric Manganese Bismuth Thiocyanate Perovskites
Source: Chem Mater. 2026 Oct 8;38(20):10805–16. doi: 10.1021/acs.chemmater.6c02127 (PMC13718557; doi:10.1021/acs.chemmater.6c02127)
Supplement: Supplementary file 1 [file cm6c02127_si_001.pdf]

Non-centrosymmetric manganese bismuth thiocyanate perovskites

Madeleine Geers[a,b,f], Dominik J. Kubicki[c], Stephen P. Argent[a], Jeremiah P. Tidey[d], Elodie Salager[g,h], Benjamin B. Duff[i], Oscar Fabelo[b], Laura Cañadillas-Delgado[b], and Matthew J. Cliffe *[a,e]

[a]School of Chemistry, University of Nottingham, University Park, Nottingham, NG7 2RD, United Kingdom
[b]Institut Laue-Langevin, 71 avenue des Martyrs CS 20156, 38042 Grenoble Cedex 9, France
[c]School of Chemistry, University of Birmingham, Birmingham B15 2TT, United Kingdom
[d]Department of Physics, University of Warwick, Gibbet Hill Road, Coventry, CV4 7AL, United Kingdom
[e]Department of Materials Science and Metallurgy, University of Cambridge, 27 Charles Babbage Road, Cambridge CB3 0FS, United Kingdom
[f]Department of Chemistry - Ångström Laboratory, Uppsala University, Box 538, 751 21, Uppsala, Sweden
[g]CNRS, Université d'Orleans, CEMHTI UPR 3079, 1D avenue de la recherche scientifique, 45071 Orleans, France
[h]RS2E, CNRS FR 3459, 15 rue Baudelocque, 80039 Amiens, France
[i]Manchester Institute of Biotechnology, University of Manchester, Manchester M13 9PL, United Kingdom

September 24, 2026

Contents

List of Figures 2

List of Tables 2

S1 Synthesis 3

S2 Powder X-ray diffraction 3

S3 Single crystal X-ray diffraction 3

S4 Single crystal 3D electron diffraction 6

*mjc222@cam.ac.uk

S5 Single crystal neutron diffraction 6

S6 MAS NMR 6

S7 Magnetometry 7

S8 Crystallographic Tables 9

List of Figures

S1 Le Bail refinement of the powder X-ray diffraction data of **KMnBi** as shown in Fig. 2(a) of the main text. . . . 3

S2 Reciprocal lattice planes of **GuaMnBi** with both the unit cell and aristotype $Pm\bar{3}m$ cubic unit cell shown. Top: $hk0$ and bottom:$h0l$. . . . 5

S3 The derivative of the susceptibility for a) **KMnBi**, b) **NH_4MnBi** and c) **GuaMnBi**. . . . 7

S4 Isothermal magnetisation measurements at 2 K for a) **KMnBi** and b) **GuaMnBi**, over an external field range of −5 to +5 T. . . . 8

S5 The derivative of the isothermal magnetisation at 2 K for a) **KMnBi** and b) **GuaMnBi**. . . 8

S6 The Mn$\{$Bi(SCN)$_6\}$ cages for **GuaMnBi** showing a) a cage with an ordered Gua$^+$ cation, and b) a cage with the Gua$^+$ cation is disordered over two orientations (red and blue atoms). Bi = purple, Mn = pink, N = blue, C = black, S = yellow, H = grey. Grey dashed lines act as a guide for the cage boundaries between Bi and Mn atoms. . . . 8

List of Tables

S1 Acquisition parameters used for the solid-state NMR data reported in the main text. . 7

S2 Single crystal experimental details for **NH_4MnBi** at 20 K (neutrons) and 120 K (X-rays). $S =$ ”Goodness of fit” . . . 9

S3 Single crystal X-ray diffraction experimental details for **CsMnBi** at 293 K and 400 K. $S =$ ”Goodness of fit” . . . 10

S4 Single crystal diffraction experimental details for **CsMnBi** at 80 K (neutrons) and 293 K after heating (X-rays). $S =$ ”Goodness of fit” . . . 11

S5 Single crystal diffraction experimental details for **GuaMnBi** (X-rays) and **KMnBi** (electrons). $S =$ ”Goodness of fit” . . . 12

S1 Synthesis

$Bi(NO_3)_3 \cdot 5\,H_2O$ (485 mg, 1 mmol) was suspended in butanone ($CH_3COC_2H_5$, 5 mL) and KNCS (583 mg, 6 mmol) was added, forming an opaque yellow-orange solution with a white precipitate. $Mn(NO_3)_2 \cdot 4\,H_2O$ (251 mg, 1 mmol) was added and the mixture was stirred overnight. The mixture was filtered and solvent removed from the yellow-orange solution *in vacuo*, yielding an orange microcrystalline powder of KMn{Bi(SCN)$_6$} (**KMnBi**).
Analogous syntheses were carried out for the other A-site cations, replacing KNCS with the appropriate thiocyanate salt. $[\square_{\frac{7}{8}}Cs(H_2O)_{\frac{1}{8}}]$Mn{Bi(SCN)$_6$} (**CsMnBi**) was synthesised from CsNCS (1146 mg, 6 mmol), $[\square_{\frac{7}{8}}NH_4(H_2O)_{\frac{1}{8}}]$Mn{Bi(SCN)$_6$} (**NH$_4$MnBi**) from NH_4NCS (457 mg, 6 mmol) and $[\square_3Gua_6]Mn_9$\{Bi(SCN)$_6$\}$_8 \cdot 6\,H_2O$ (**GuaMnBi**) from GuaNCS (709 mg, 6 mmol). The synthesis of **NH$_4$MnBi** for X-ray diffraction measurements and **CsMnBi** were carried out using acetone (CH_3COCH_3) as the solvent.
Single crystals of **NH$_4$MnBi**, **CsMnBi** and **GuaMnBi** were obtained by slow evaporation recrystallisations from their mother liquor for a duration of 2 to 7 months.

S2 Powder X-ray diffraction

Powder X-ray diffraction data were collected for **KMnBi** using a Panalytical Empyrean X-ray diffractometer equipped with GaliPIX3D area detector and a sealed tube Cu source ($\lambda = 1.542$ Å) operating at 40 kV and 40 mA. The sample was prepared between two layers of Kapton film. Data were collected with a step size of $0.02°$ in continuous mode and a scan speed of 15 seconds per step. Repeated scans were measured and merged after inspection to ensure no sample degradation had occurred. Structural refinements were carried out using the FullProf program.[1]

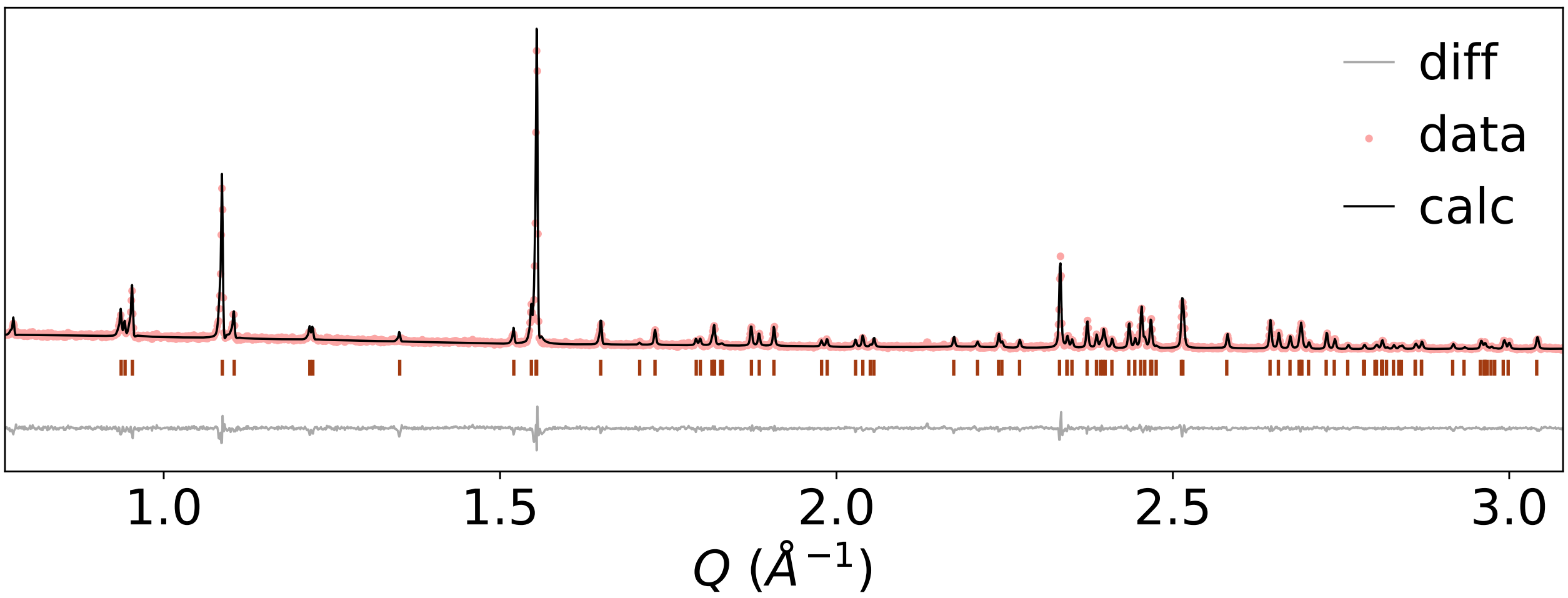

Figure S1: Le Bail refinement of the powder X-ray diffraction data of **KMnBi** as shown in Fig. 2(a) of the main text.

S3 Single crystal X-ray diffraction

Single crystal X-ray diffraction was carried out for **NH$_4$MnBi** and **GuaMnBi** using an Oxford Diffraction GV1000 (AtlasS2 CCD area detector, mirror-monochromated Mo-Kα radiation source; $\lambda = 0.71073$ Å; ω scans). Measurements were carried out at 120 K by a stream of cold N_2 using an Oxford Cryosystems open flow cryostat.[2] Cell parameters were refined from the observed positions of all strong

reflections and absorption corrections were applied using a Gaussian numerical method with beam profile correction (CrysAlisPro).[3]
The structures were solved within Olex2[4] by dual space iterative methods (SHELXT),[5] and least squares refinement of the structural models were carried using SHELXL.[6] Complete experimental and refinement information are contained within the deposited CIF along with structure factors in the .fcf; the CIF are deposited in the ICSD with Deposition Numbers CSD 2571827 and 2573105.
Single crystal X-ray measurements were carried out for **CsMnBi** at 293 K, 400 K, and 293 K (after 400 K measurement) using a Bruker D8 Venture diffractometer. A graphite monochromatic radiation source from Ag-Kα radiation, $\lambda = 0.56089$ Å was used. Cell parameters were refined from the observed positions of all strong reflections and absorption corrections were applied using a Gaussian numerical method with beam profile correction (APEX2).[7] Structure solution for **CsMnBi** was carried out using *SUPERFLIP*[8] and refined using Jana2006.[9] Complete experimental and refinement information are contained within the deposited CIF along with structure factors in the .fcf; the CIF are deposited in the ICSD with Deposition Numbers CSD 2571899, 2571867 and 2573101.

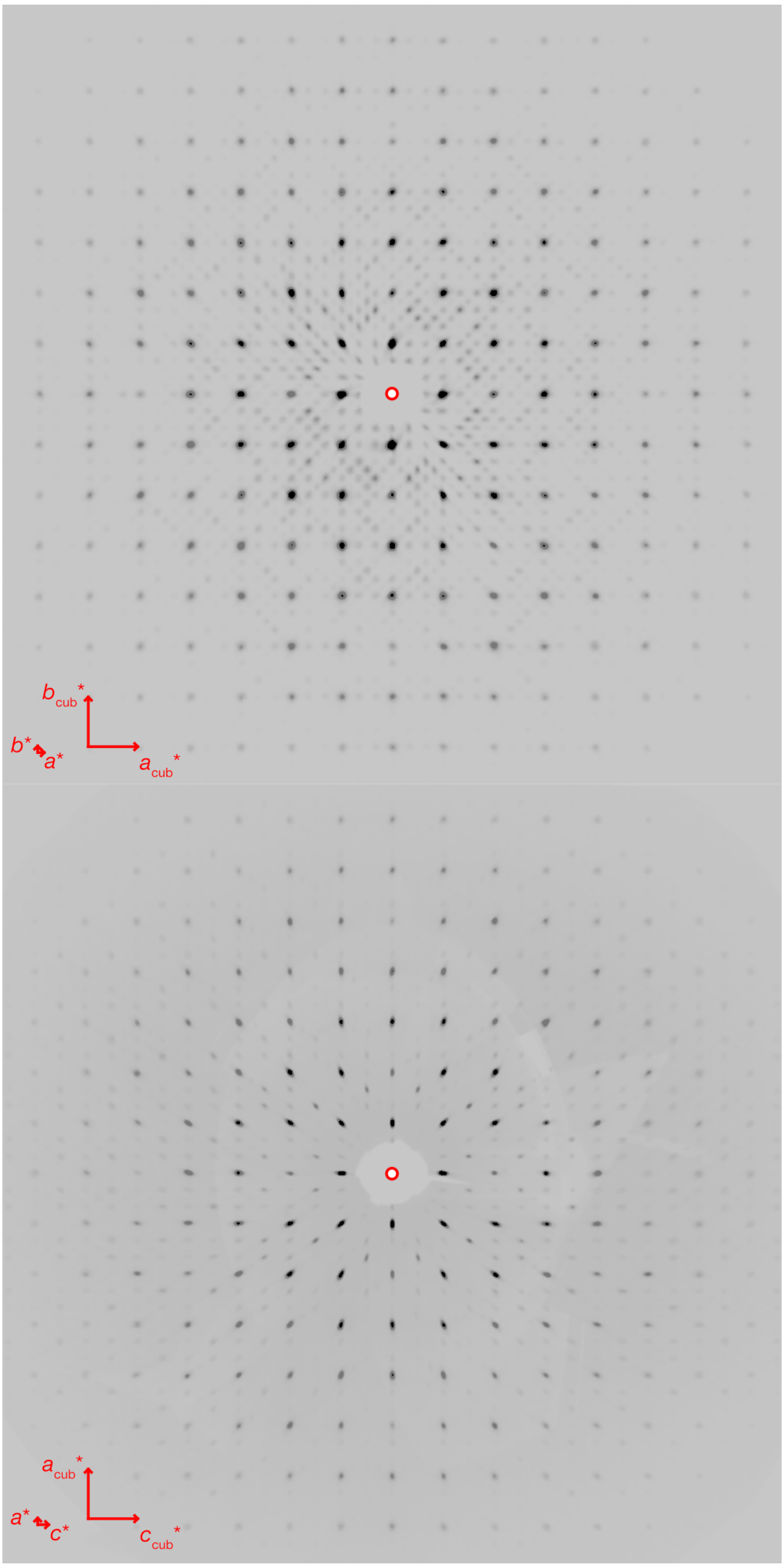

Figure S2: Reciprocal lattice planes of **GuaMnBi** with both the unit cell and aristotype $Pm\bar{3}m$ cubic unit cell shown. Top: $hk0$ and bottom:$h0l$.

S4 Single crystal 3D electron diffraction

Microcrystalline powder of **KMnBi** was lightly ground and dispersed dry onto a copper-supported continuous amorphous carbon TEM grid and loaded at room temperature *via* a JEOL high-tilt specimen holder into a Rigaku XtaLAB Synergy-ED electron diffractometer, operated at 200 kV and equipped with a Rigaku HyPix-ED hybrid pixel array area detector. Data were collected on various crystallites as single-rotation scans collecting 0.5° frames using *CrysAlisPRO* system (CCD 1.171.43.62a 64-bit (release 17-02-2023))[10] using continuous rotation electron diffraction with a selected area aperture of either 2 or 4 µm apparent diameter. Further experimental details are provided in Table S5.
All single crystal component datasets were individually indexed and integrated, prior to the merging of four suitable datasets for scaling, using *CrysAlisPRO* (version 1.171.44.144a)[10]; no absorption corrections were applied. Data were integrated and merged to a resolution limit of 0.92 Å. The structure was solved using ShelXT[5] and refined using Olex2. The structure was refined in the kinematic approximation, applying an extinction correction to broadly account for multiple scattering, alongside rejection of clearly outlying reflections, as implemented in Olex2 (version 1.5-ac7-022, compiled 2026.02.09 svn.ra45a96f9 for Rigaku Oxford Diffraction, GUI svn.r7493)[4,11] and using published scattering factors.[12]
All non-H atoms are refined with anisotropic displacement parameters with a global rigid bond restraint, proximal similarity restraints across each NCS unit, and further isotropicity restraints applied to improve the physical sense of the ADPs. 1,2 and 1,3 distances in NCS units are restrained to refine equally to provide chemically reasonable geometry. Complete experimental and refinement information are contained within the deposited CIF along with structure factors and embedded .RES file; CIF deposited in the ICSD with Deposition Number CSD 2466903.

S5 Single crystal neutron diffraction

Monochromatic single crystal neutron diffraction data for **NH_4MnBi** and **CsMnBi** were collected on the four-circle D19 diffractometer (ILL). Neutrons with a wavelength of 1.458 Å were used, provided by a flat Cu monochromator using the 220 reflection at $2\theta_M = 69.91$° take-off angle. The samples were placed in a closed-circuit displex cooling device, which was operated following a ramp of 2 K min^{-1}. Measurements were carried out at 20 K (**NH_4MnBi**) and 80 K (**CsMnBi**), using NOMAD software for data collection.[13]
Unit cell determinations were performed using PFIND and DIRAX programs, and processing of the raw data was applied using RETREAT, RAFD19 and Int3D programs.[14–17] The data were corrected for the absorption of the low-temperature device using the D19ABSCAN program.[18] The structures were solved using *SUPERFLIP*[8] and refined using Jana2006.[9] For **NH_4MnBi**, bond distance restraints were applied to N14−H5,6,7 and N15−H10, and bond angle restraints for H5−N14−H7 to physically meaningful values. In addition, a riding model with tetrahedral geometry was applied to add H9,11,12, using N15−H10 as an anchor. Raw datasets from ILL experiments can be accessed via links provided in the references.[19,20] Complete experimental and refinement information are contained within the deposited CIF along with structure factors in the .fcf file; the CIF are deposited in the ICSD with Deposition Number CSD 2571818 and 2571841.

S6 MAS NMR

Samples were packed into 1.3 mm (for 4.7 T experiments) or 3.2 mm (for 100 K measurements at 9.4 T) zirconia rotors. Solid-state MAS NMR spectra of ^{1}H (200.15 MHz), ^{13}C (50.32 MHz) and ^{133}Cs (26.25 MHz) were recorded on a Bruker Avance III 4.7 T spectrometer using 167 kHz and 111 RF field, respectively. Variable-temperature ^{133}Cs MAS spectra were recorded using the same setup. ^{1}H MAS at 100 K were recorded using a 3.2 mm LTMAS probe and an RF strength of 100 kHz. Chemical shifts were referenced to solid adamantane using the IUPAC convention (^{13}C at 38.48 ppm for the CH_2

carbon, and using the respective ratios of gyromagnetic ratios for ^{1}H and ^{133}Cs). Further experimental details (spin rates, measured T_1 values, recycle delays, pulse programmes) are reported in Table S1.

Table S1: Acquisition parameters used for the solid-state NMR data reported in the main text.

Sample	Nucleus	T (K)	Spin rate (kHz)	Field (T)	Fig.	Pulse sequence	T_1 (s)	Recycle delay (s)	Scans	Exp. time (h)
CsMnBi	^{1}H	300	30	4.7	5a	echo	0.4	2.5	92	0.06
NH_4MnBi	^{1}H	300	20	4.7	5a	echo	4.6	25	16	0.11
NH_4MnBi	^{1}H	100	8	9.4	5a	echo	0.5	0.1	1024	0.03
GuaMnBi	^{1}H	300	20	4.7	5a	echo	2	10	32	0.09
GuaMnBi	^{13}C	300	8	4.7	5a	echo	n/d	1	49952	13.9
CsMnBi	^{133}Cs	300	40	4.7	5b	echo	< 0.005	0.005	44120	0.06
CsMnBi	^{133}Cs	283	40	4.7	5b	echo	< 0.005	0.005	30000	0.04
CsMnBi	^{133}Cs	273	40	4.7	5b	echo	< 0.005	0.005	40000	0.06
CsMnBi	^{133}Cs	263	40	4.7	5b	echo	< 0.005	0.002	80000	0.04
CsMnBi	^{133}Cs	253	40	4.7	5b	echo	< 0.005	0.005	80000	0.11

S7 Magnetometry

Measurements of the magnetic susceptibility were carried out for **KMnBi**, **NH_4MnBi** and **GuaMnBi** using a Quantum Design Magnetic Property Measurements System (MPMS) 3 Superconducting Quantum Interference Device (SQUID) magnetometer. The zero-field-cooled and field-cooled susceptibility was measured in an applied field of 0.01 T over the temperature range $2-300$ K. As *M*(H) is linear in this field, the small-field approximation for the susceptibility, $\chi(T) \simeq \frac{M}{H}$, where *M* is the magnetisation and *H* is the magnetic field intensity, was taken to be valid. Isothermal magnetisation measurements were carried out for **KMnBi** and **GuaMnBi** at 2 K over the field range -5 to $+5$ T. Data were corrected for diamagnetism of the sample using Pascal's constants.[21]

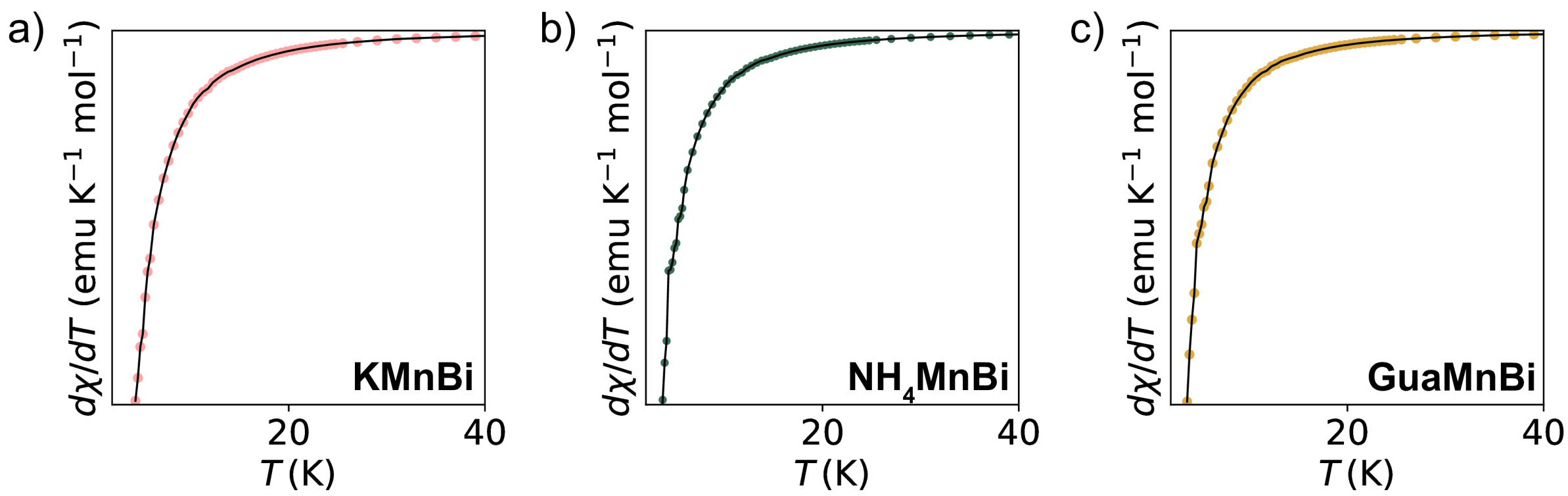

Figure S3: The derivative of the susceptibility for a) **KMnBi**, b) **NH_4MnBi** and c) **GuaMnBi**.

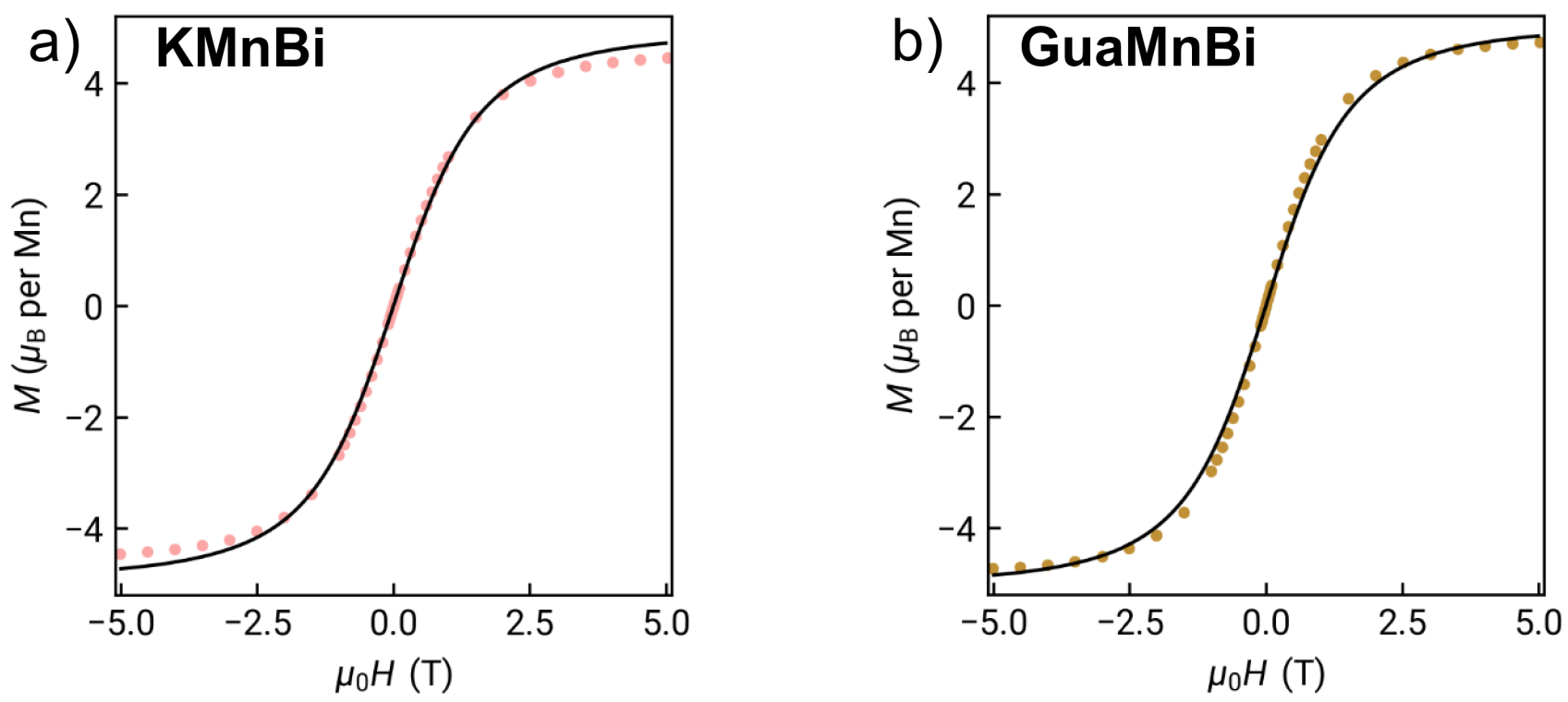

Figure S4: Isothermal magnetisation measurements at 2 K for a) **KMnBi** and b) **GuaMnBi**, over an external field range of −5 to +5 T.

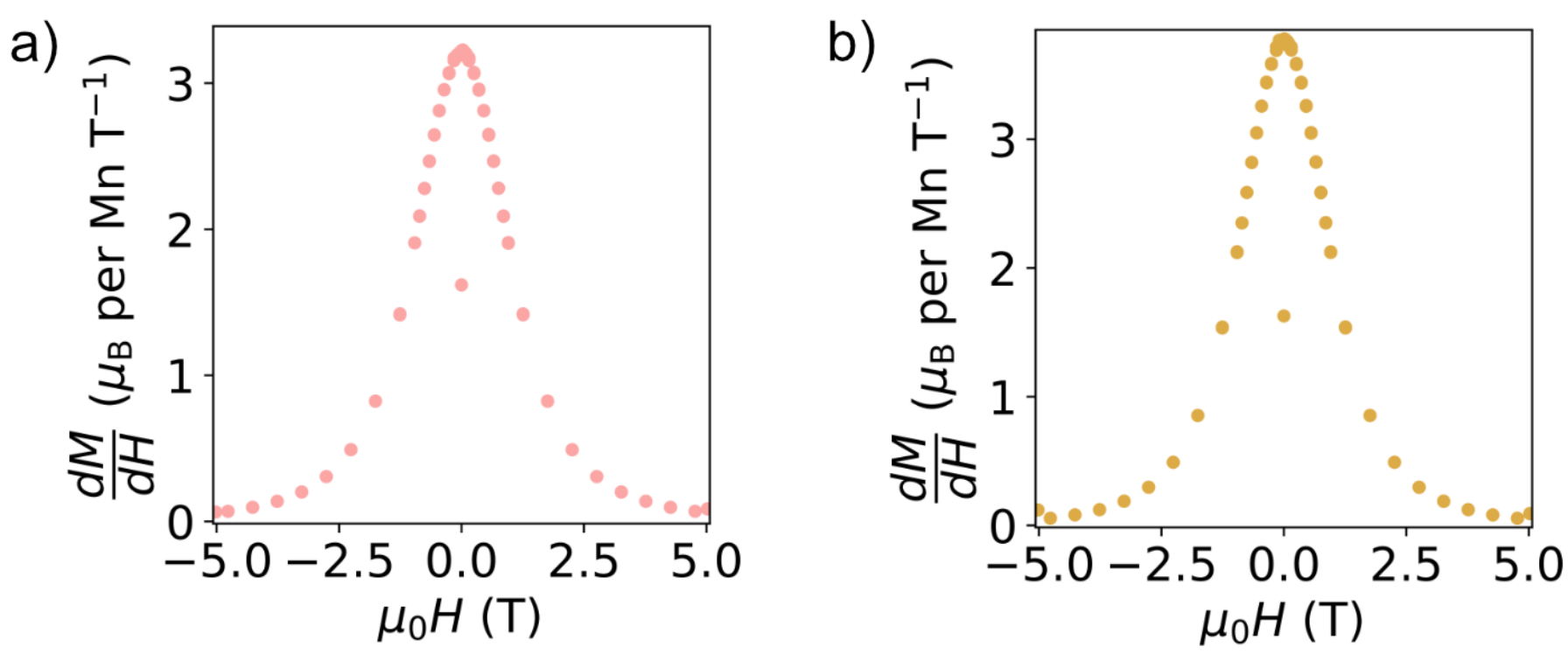

Figure S5: The derivative of the isothermal magnetisation at 2 K for a) **KMnBi** and b) **GuaMnBi**.

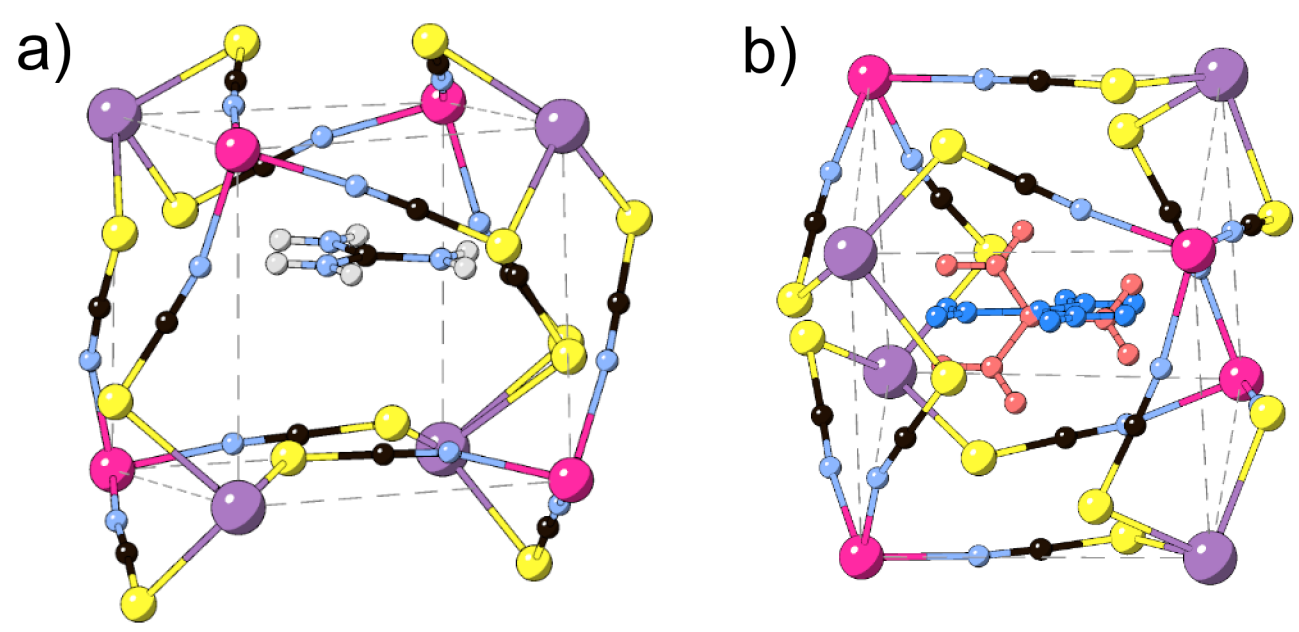

Figure S6: The Mn{$Bi(SCN)_6$} cages for **GuaMnBi** showing a) a cage with an ordered Gua^+ cation, and b) a cage with the Gua^+ cation is disordered over two orientations (red and blue atoms). Bi = purple, Mn = pink, N = blue, C = black, S = yellow, H = grey. Grey dashed lines act as a guide for the cage boundaries between Bi and Mn atoms.

S8 Crystallographic Tables

Table S2: Single crystal experimental details for **NH_4MnBi** at 20 K (neutrons) and 120 K (X-rays). $S =$ "Goodness of fit"

Chemical formula	$C_{48}H_{28}Bi_8Mn_8N_{56}OS_{48}$	$Bi_8C_{48}Mn_8N_{56}S_{48}O_1$
M_r	5055.4	5027.28
Temperature (K)	20(2)	120(2)
λ (Å)	1.45818	0.7107
Radiation type	Neutron	Mo-$K\alpha$ X-rays
Crystal system	Orthorhombic	Orthorhombic
Space group	$Pnnn$	$Pnnn$
a (Å)	12.442(3)	12.4172(3)
b (Å)	24.422(3)	24.4213(6)
c (Å)	24.3705(17)	24.5217(7)
V (Å^3)	7405(2)	7436.1(3)
Z	2	2
μ (mm^{-1})	0.01	10.81
Crystal size (mm)	$3 \times 3 \times 1$	$0.14 \times 0.07 \times 0.03$
Data collection		
Diffractometer	D19 (ILL)	GV1000, AtlasS2
No. of measured reflections	24833	236766
No. of independent reflections	6435	19490
No. of observed $[I > 3\sigma(I)]$ reflections	2556	8361
R_{int}	0.247	0.128
$(\sin\theta/\lambda)_{max}$ (Å^{-1})	0.599	0.863
Data completeness	0.965	0.971
Refinement		
$R[F^2 > 3\sigma(F^2)]$	0.160	0.084
$wR(F^2)$	0.161	0.102
S	3.120	2.37
No. of parameters	390	384
$\Delta\rho >_{max}$, $\Delta\rho >_{min}$ (e Å^{-3})	+4.82, −2.79	+2.35, −1.14

Table S3: Single crystal X-ray diffraction experimental details for **CsMnBi** at 293 K and 400 K. S = "Goodness of fit"

Chemical formula	$C_{96}Bi_{16}Cs_{16}Mn_{16}N_{96}O_2S_{96}$	$C_{48}Bi_8Cs_8Mn_8N_{48}S_{48}$
M_r	11956.7	5962.3
Temperature (K)	293(2)	400(2)
λ (Å)	0.56088	0.56089
Radiation type	Ag-$K\alpha$ X-rays	Ag-$K\alpha$ X-rays
Crystal system	Orthorhombic	Orthorhombic
Space group	$Pnnn$	$Pnnn$
a (Å)	12.4386(4)	12.452(4)
b (Å)	24.7803(7)	24.936(9)
c (Å)	24.8038(7)	24.859(9)
V (Å^3)	7645.3(4)	7719(5)
Z	1	2
μ (mm^{-1})	6.70	6.64
Crystal size (mm)	$0.1 \times 0.08 \times 0.05$	$0.1 \times 0.08 \times 0.05$
Data collection		
Diffractometer	Bruker D8 Venture	Bruker D8 Venture
No. of measured reflections	235994	74776
No. of independent reflections	18782	11738
No. of observed $[I > 3\sigma(I)]$ reflections	8672	4429
R_{int}	0.080	0.090
(sin $\theta/\lambda)_{max}$ (Å^{-1})	0.837	0.716
Data completeness	1.000	0.986
Refinement		
$R[F^2 > 2\sigma(F^2)]$	0.0610	0.091
$wR(F^2)$	0.0814	0.169
S	2.13	3.10
No. of parameters	405	380
$\Delta\rho >_{max}, \Delta\rho >_{min}$ (e Å^{-3})	$+8.52, -4.48$	$+10.32, -4.70$

Table S4: Single crystal diffraction experimental details for **CsMnBi** at 80 K (neutrons) and 293 K after heating (X-rays). $S =$ "Goodness of fit"

Chemical formula	$C_{96}Bi_{16}Cs_{16}Mn_{16}N_{96}O_2S_{96}$	$C_{96}Bi_{16}Cs_{16}Mn_{16}N_{96}S_{96}$
M_r	11940.7	11924.96
Temperature (K)	80(2)	293(2)
λ (Å)	1.45947	0.56089
Radiation type	Neutron	Ag-$K\alpha$ X-rays
Crystal system	Orthorhombic	Orthorhombic
Space group	$Pnnn$	$Pnnn$
a (Å)	12.3953(4)	12.4386(3)
b (Å)	24.6320(7)	24.7803(8)
c (Å)	24.6136(7)	24.8038(8)
V (Å^3)	7515.0(4)	7645.3(4)
Z	1	1
μ (mm^{-1})	0.01	6.7
Crystal size (mm)	$4 \times 3 \times 2$	$0.1 \times 0.08 \times 0.05$
Data collection		
Diffractometer	D19 (ILL)	Bruker D8 Venture
No. of measured reflections	17289	155027
No. of independent reflections	6154	11747
No. of observed $[I > 3\sigma(I)]$ reflections	4063	7413
R_{int}	0.077	0.069
(sin $\theta/\lambda)_{max}$ (Å^{-1})	0.598	0.716
Data completeness	0.913	0.997
Refinement		
$R[F^2 > 3\sigma(F^2)]$	0.251	0.067
$wR(F^2)$	0.267	0.223
S	8.86	1.03
No. of parameters	371	389
$\Delta\rho >_{max}$, $\Delta\rho >_{min}$ (e Å^{-3})	$+2.99$, -2.35	$+8.14$, -2.82

Table S5: Single crystal diffraction experimental details for **GuaMnBi** (X-rays) and **KMnBi** (electrons). $S =$ "Goodness of fit"

Chemical formula	$C_{56}H_{48}Bi_8Mn_9N_{72}O_6S_{48}$	$BiC_6KMnN_6S_6$
M_r	5530.84	651.52
Temperature (K)	$120(2)$	$293(5)$
λ (Å)	0.7107	0.0251
Radiation type	Mo-$K\alpha$ X-rays	Electron
Crystal system	Tetragonal	Monoclinic
Space group	$I\bar{4}2d$	Pn
a (Å)	$37.0950(4)$	$8.715(3)$
b (Å)	$37.0950(4)$	$8.551(3)$
c (Å)	$24.4182(4)$	$12.005(4)$
β (°)	90	$90.25(3)$
V (Å^3)	$33600.4(9)$	$894.6(6)$
Z	8	2
μ (mm^{-1})	9.65	0
Crystal size (mm)	$0.13 \times 0.11 \times 0.09$	approx. $0.001 \times 0.001 \times 0.001$
Data collection		
Diffractometer	GV1000, AtlasS2	XtaLAB Synergy-ED, HyPix-ED, electron source at 200 keV
No. of measured reflections	318851	7434
No. of independent reflections	17173	2181
No. of observed $[I > 2\sigma(I)]$ reflections	14689	1207
R_{int}	0.077	0.321
(sin $\theta/\lambda)_{max}$ (Å^{-1})	0.625	0.542
Data completeness	0.999	0.997
Refinement		
$R[F^2 > 2\sigma\ (F^2)]$	0.055	0.131
$wR(F^2)$	0.135	0.316
S	1.10	1.04
No. of parameters	870	191
$\Delta\rho >_{max},\ \Delta\rho >_{min}$ (e Å^{-3})	$+3.12,\ -1.82$	$+2.92,\ -1.46$

References

[1] J. Rodríguez-Carvajal, *Physica B: Condensed Matter*, 1993, **192**, 55–69.
[2] J. Cosier and A. M. Glazer, *Journal of Applied Crystallography*, 1986, **19**, 105–107.
[3] Rigaku Oxford Diffraction, *CrysAlisPro Software system, version 1.171.40.45a*, 2018.
[4] O. V. Dolomanov, L. J. Bourhis, R. J. Gildea, J. A. K. Howard and H. Puschmann, *Journal of Applied Crystallography*, 2009, **42**, 339–341.
[5] G. M. Sheldrick, *Acta Crystallographica Section A: Foundations of Crystallography*, 2015, **71**, 3–8.
[6] G. M. Sheldrick, *Acta Crystallographica Section C Structural Chemistry*, 2015, **71**, 3–8.
[7] Bruker, *APEX2 version 2.0-22*, 2004.
[8] L. Palatinus and G. Chapuis, *Journal of Applied Crystallography*, 2007, **40**, 786–790.
[9] V. Petříček, M. Dušek and L. Palatinus, *Zeitschrift für Kristallographie-Crystalline Materials*, 2014, **5**, 345–352.
[10] Rigaku Oxford Diffraction, *CrysAlisPro Software system*, 2025.
[11] L. J. Bourhis, O. V. Dolomanov, R. J. Gildea, J. A. K. Howard and H. Puschmann, *Acta Crystallographica Section A Foundations and Advances*, 2015, **71**, 59–75.
[12] A. Saha, S. S. Nia and J. A. Rodríguez, *Chemical Reviews*, 2022, **122**, 13883–13914.
[13] P. Mutti, F. Cecillon, A. Elaazzouzi, Y. Le Goc, J. Locatelli, H. Ortiz and J. Ratel, Proc. ICALEPCS, 2011.
[14] A. J. M. Duisenberg, *Journal of Applied Crystallography*, 1992, **25**, 92–96.
[15] G. J. McIntyre and R. F. D. Stansfield, *Acta Crystallographica Section A Foundations of Crystallography*, 1988, **44**, 257–262.
[16] C. Wilkinson, H. W. Khamis, R. F. D. Stansfield and G. J. McIntyre, *Journal of Applied Crystallography*, 1988, **21**, 471–478.
[17] N. A. Katcho, L. Cañadillas-Delgado, O. Fabelo, M. T. Fernández-Díaz and J. Rodríguez-Carvajal, *Crystals*, 2021, **11**, 897.
[18] J. C. Matthewman, P. Thompson and P. J. Brown, *Journal of Applied Crystallography*, 1982, **15**, 167–173.
[19] L. Cañadillas-Delgado, M. J. Cliffe, O. Fabelo and M. Geers, *ILL experiment 5-15-633*, `http://doi.ill.fr/10.5291/ILL-DATA.5-15-633`.
[20] L. Cañadillas-Delgado, M. J. Cliffe, O. Fabelo and M. Geers, *ILL experiment 5-15-633*, `http://doi.ill.fr/10.5291/ILL-DATA.EASY-1253`.
[21] G. A. Bain and J. F. Berry, *Journal of Chemical Education*, 2008, **85**, 532.
